# Pentacyclic adenine: a versatile and exceptionally bright fluorescent DNA base analogue†
†Electronic supplementary information (ESI) available. See DOI: 10.1039/c7sc05448c


**DOI:** 10.1039/c7sc05448c

**Published:** 2018-03-01

**Authors:** Mattias Bood, Anders F. Füchtbauer, Moa S. Wranne, Jong Jin Ro, Sangamesh Sarangamath, Afaf H. El-Sagheer, Déborah L. M. Rupert, Rachel S. Fisher, Steven W. Magennis, Anita C. Jones, Fredrik Höök, Tom Brown, Byeang Hyean Kim, Anders Dahlén, L. Marcus Wilhelmsson, Morten Grøtli

**Affiliations:** a Department of Chemistry and Molecular Biology , University of Gothenburg , SE-412 96 Gothenburg , Sweden . Email: grotli@chem.gu.se; b Department of Chemistry and Chemical Engineering, Chemistry and Biochemistry , Chalmers University of Technology , SE-412 96 Gothenburg , Sweden . Email: marcus.wilhelmsson@chalmers.se; c Chemistry Branch , Faculty of Petroleum and Mining Engineering , Suez University , Suez 43721 , Egypt; d WestCHEM , School of Chemistry , University of Glasgow , Glasgow , G12 8QQ , UK; e School of Chemistry , University of Edinburgh , The King's Buildings , Edinburgh EH9 3JJ , UK; f Department of Chemistry , Division of Advanced Materials Science , Pohang University of Science and Technology (POSTECH) , Pohang 37673 , South Korea; g Department of Chemistry , Chemistry Research Laboratory , University of Oxford , Oxford , OX1 3TA , UK; h Division of Biological Physics , Department of Physics , Chalmers University of Technology , SE-412 96 Gothenburg , Sweden; i AstraZeneca R&D , Innovative Medicines , Cardiovascular & Metabolic Diseases (CVMD) , Pepparedsleden 1, SE-431 83 Mölndal , Gothenburg , Sweden

## Abstract

Emissive base analogs are powerful tools for probing nucleic acids at the molecular level. Herein we describe the development and thorough characterization of pentacyclic adenine (pA), a versatile base analog with exceptional fluorescence properties. When incorporated into DNA, pA pairs selectively with thymine without perturbing the B-form structure and is among the brightest nucleobase analogs reported so far. Together with the recently established base analog acceptor qA_nitro_, pA allows accurate distance and orientation determination *via* Förster resonance energy transfer (FRET) measurements. The high brightness at emission wavelengths above 400 nm also makes it suitable for fluorescence microscopy, as demonstrated by imaging of single liposomal constructs coated with cholesterol-anchored pA–dsDNA, using total internal reflection fluorescence microscopy. Finally, pA is also highly promising for two-photon excitation at 780 nm, with a brightness (5.3 GM) that is unprecedented for a base analog.

## Abstract 2

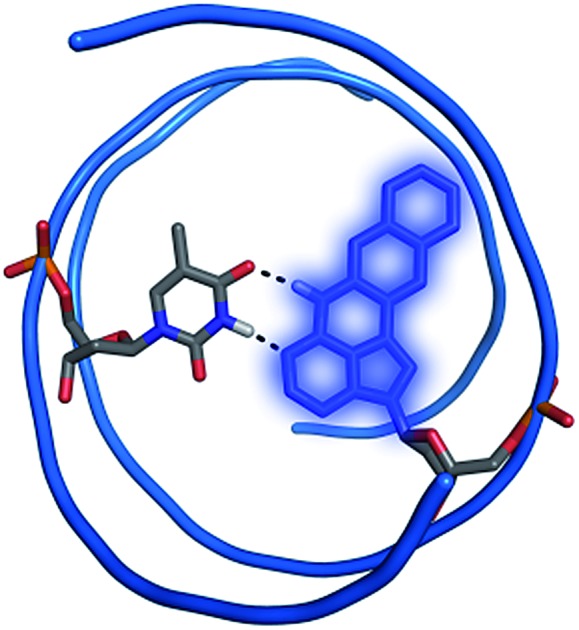
A highly fluorescent, non-perturbing, pentacyclic adenine analog was designed, synthesized, incorporated into DNA and photophysical evaluated.

## Introduction

Fluorescent base analogues (FBAs) are emissive structural mimics of the canonical nucleic acid bases that retain the ability to form Watson–Crick hydrogen bonds with their complementary bases. With their position inside the nucleic acid they offer several advantages compared to external, covalently attached fluorophores, *e.g.* lower bulkiness, possibility of labelling close to a site of interest, lower interference during interaction between the nucleic acid and other molecules and higher precision in position as well as orientation. FBAs have been used successfully for microenvironment sensing and have become powerful tools for studying the structure and dynamics of nucleic acids.^1^–^5^ Recent developments include a thymidine mimic used for site-specific metal binding in dsDNA,^6^,^7^ a tricyclic cytidine analogue with a turn-on response to duplex formation,^8^ isomorphic emissive RNA derivatives,^9^,^10^ a novel quadracyclic adenosine Förster resonance energy transfer (FRET) pair^11^ and an emissive interbase FRET pair.^12^

**Chart 1:**
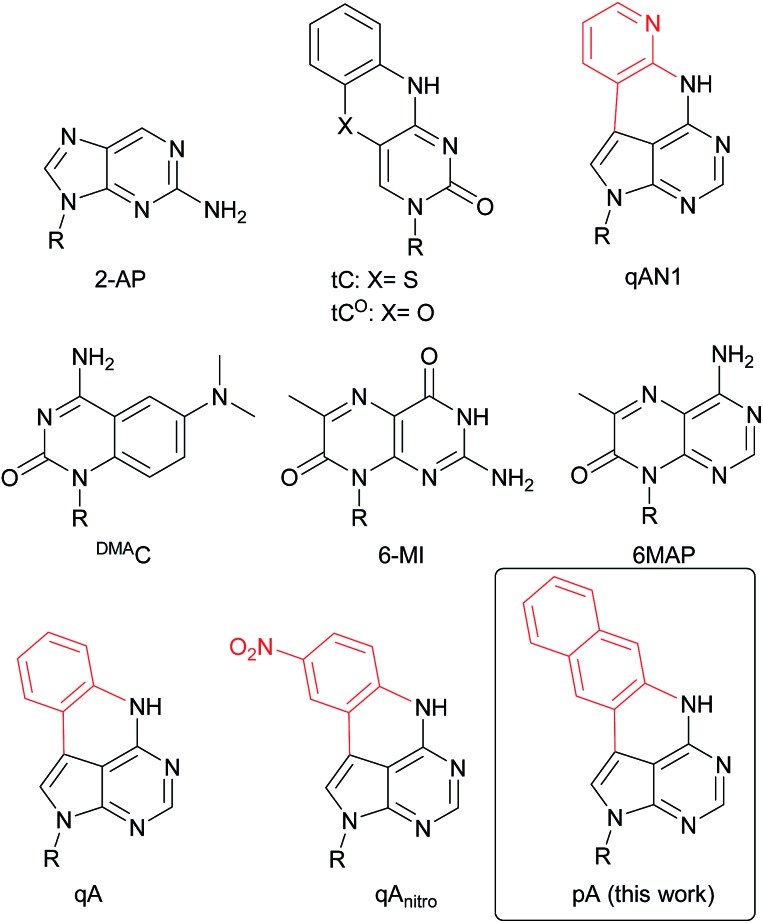
Structure of 2-AP, tC, tC^O^, qAN1, ^DMA^C, 6-MI, 6MAP, qA, qA_nitro_ and pA. R denotes the sugar–phosphate backbone. Atoms of extended adenine (with N-7 replaced with C) are shown in red.

Generally, the incorporation of FBAs into oligonucleotides results in a significant decrease in fluorescence intensity, even more so upon hybridization to complementary strands. For example, the brightness of 2-aminopurine (2-AP, Chart 1) drops almost 100-fold upon incorporation in dsDNA.^16^,^17^ However, with a few exceptions (*e.g.* tC,^13^,^14^ tC^O^,^15^ qAN1,^11^Chart 1), quantum yields and normal (one-photon) brightness values of FBAs inside oligonucleotides are characterized only for a few sequence contexts, which limits a comparison between them (for a detailed comparison of one- and two-photon emissive properties of FBAs, see Table 1). A few probes essentially retain (tC^O^) or even exhibit enhanced fluorescence quantum yield and brightness (*e.g.* tC and ^DMA^C, Chart 1) in dsDNA.^13^,^14^,^18^ Still, these fluorophores are significantly less bright than the most commonly used external fluorophores (Cy-, Alexa-, ATTO-dyes). For detailed real-time information on the structure and intrinsic dynamics of nucleic acids,^19^,^20^ as well as their sub-cellular or cellular location, FBAs with a lower detection limit would be advantageous. In particular, FBAs that are bright enough, and sufficiently resistant to photobleaching, are required for single-molecule studies and super-resolution imaging.^21^ A few groups have investigated single-molecule detection of FBAs, however, with limited success. For example, 3-MI monomers were shown by fluorescence correlation spectroscopy to have a brightness of 4 kHz per molecule and a signal-to-background (S/B) of 5, whereas the values for 3-MI-containing oligonucleotides were reduced by a factor of 4.^22^

**Table 1:** Photophysical properties of pA (this work) and previously reported FBAs^*a*^

Name	One-photon excitation					Two-photon excitation^*b*^		
	*λ*_Abs_ (nm)	*λ*_Em_ (nm)	*Φ*_F_ ^*c*^ (%)	Brightness, *εΦ*_F_ (M^–1^ cm^–1^)		*λ*_Ex_ (nm)	*σ* ^2^ ^*c*^ (GM)	*Φ*_F_ *σ* ^2^ (GM)^*d*^
				Monomer	dsDNA^*e*^			
pA	387	420	66	10 100	1400	780	6.6	5.3
qAN1 (ref. 11)	354	430	18	1700	510	740^*f*^	0.82^*f*^	0.15^*f*^
6-MI^25^,^28^	350	431	70	8400^*g*^	1700	700	2.5 39	1.8
6MAP^22^,^26^	330	430	39	3300	150	659	3.4 25	1.3
2-AP^16^,^17^	303	370	68	4080	50	584	0.2 23	0.14
8-vdA^40^	290	382	65	8200	200	n.d.	n.d.	n.d.
tC^13^,^14^	377	513	13	520	760	800	1.5^*h*^ 23	0.32
tC^O^ 15	360	465	30	2700	2000	n.d.	n.d.	n.d.
FDT^18^	316	434	3	330	n.d.	690	2.1 27	0.063
^DMA^C^41^	365	526	3	80	150	n.d.	n.d.	n.d.
^th^G^9^,^42^	321	453	46	1900	310	n.d.	n.d.	n.d.
^th^U^9^,^43^	304	409	41	1300	90^*i*^	690	0.17 27	0.070
TPAU^44^	332	455	20	2200	n.d.	690	3.8 27	0.76
ADQ^45^	316	363	4	470	n.d.	690	1.8 27	0.070

^*a*^For structures and names see Chart S1.

^*b*^Two-photon excitation determined for FBA monomers.

^*c*^Quantum yield determined in various buffered water solutions at either pH 7.0 or pH 7.5 (top 8 entries) or deionized water (bottom 6 entries).

^*d*^Goeppert-Mayer units, 1 GM = 10^–50^ cm^4^ s per photon.

^*e*^Average over various DNA sequence surroundings. However, in some cases only one sequence was reported.

^*f*^Values from ongoing studies (manuscript in preparation).

^*g*^The molar absorptivity has not been reported, but is estimated to 12 000 M^–1^ cm^–1^.^46^

^*h*^Measured in the sequence 5′-AATCTCACAGC(tC)TGATCACATTGCTA-3′.

^*i*^Quantum yield based on ^th^dT dsDNA (2.7%) in the sequence 5′-GCGCGA(^th^dT)A(^th^dT)A(^th^dT)AGGAGC-3′.^41^

Recently, there has been a growing interest in the use of two-photon absorption processes in combination with FBAs. Ultraviolet light (<400 nm), which is normally required to excite FBAs, is prone to cause photobleaching, generate high levels of background fluorescence and damage biological samples.^23^ These problems can be avoided by the use of two-photon excitation. The near-infrared light used for this process can reduce out-of-focus photobleaching and autofluorescence, in turn allowing deeper tissue penetration, and increased three-dimensional resolution.^24^

Overall, the number of FBAs investigated for two-photon excitation purposes is scarce. Among the established FBAs, 6-MI and 6MAP (Chart 1) have the highest two-photon absorption cross-sections (Table 1).^25^,^26^ Recently, the two-photon-induced fluorescence properties of several new uridine FBAs (*e.g.*^th^U, TPAU and ADQ, Table 1) have been studied.^27^ TPAU has the highest two-photon cross-section reported so far for a FBA (7.6 GM at 690 nm). However, it has a very low quantum yield (1%), resulting in a low two-photon brightness (two-photon cross-section × quantum yield). 6-MI therefore remains the FBA with the highest reported two-photon brightness (Table 1), but its quantum yield is reduced significantly inside base stacks (for a purine-rich and a pyrimidine-rich sequence: 96% and 64% quenching, respectively), which hampers its use as a two-photon probe.^28^ While advances in recent years have brought the brightness of FBAs much closer to that of external fluorophores, there is still a significant need for the development of FBAs with even higher one-photon brightness and a significantly growing interest in FBAs with two-photon brightness values high enough for practical application as two-photon probes.

Herein, we report the synthesis and characterization of pentacyclic adenine, pA, a bright and photophysically versatile fluorescent adenine analogue, and establish its usefulness for both (one-photon) FRET and two-photon purposes. Finally, pA is demonstrated to be a promising internal label for microscopy applications in a total internal reflection fluorescence (TIRF) microscopy study visualizing single liposome constructs coated with pA-containing DNA.

## Results and discussion

### Design and synthesis of pA

We have recently shown that the brightness of the promising quadracyclic adenine (qA) analogue, can be significantly improved by replacing the outer ring with a pyridine ring (Chart 1), and demonstrated the use of one of these analogues, qAN1 (Chart 1), for interbase FRET in dsDNA.^11^,^29^ A screen of pentacyclic adenine analogue motifs, wherein the outer ring of the quadracyclic scaffold is replaced with coumarins, quinolines and keto-functionalized naphthalenes (Chart S2†), was performed. After extensive synthetic efforts and photophysical characterization (data not shown), a non-substituted naphthalene scaffold (Chart 1, right) was found to have the most desirable photophysical properties.

**Scheme 1:**
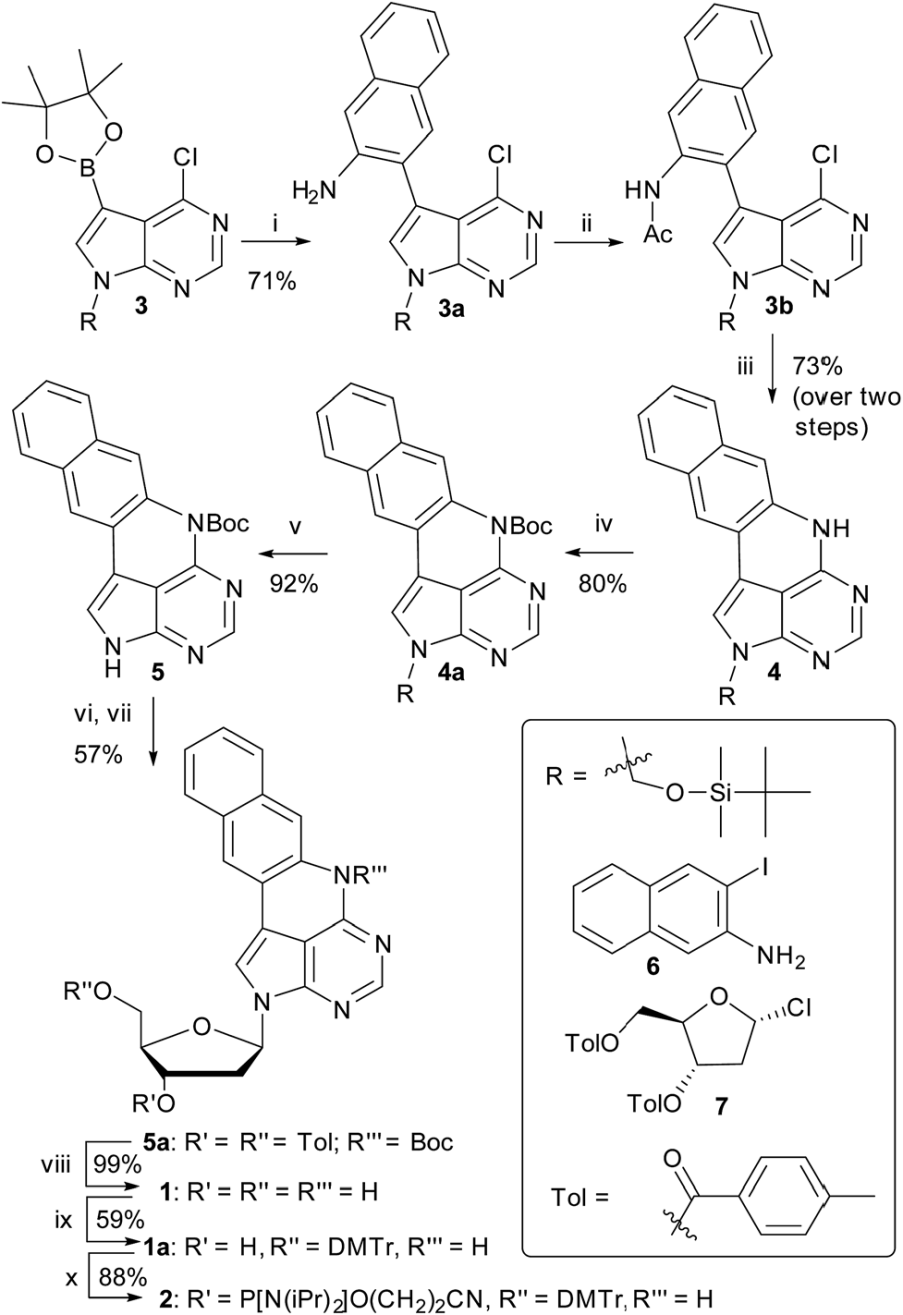
Synthesis of the pA phosphoramidite. Reagents and conditions: (i) **6** (1 equiv.) Pd(PPh_3_)_2_Cl_2_ (4.4 mol%), K_2_CO_3_ (2.5 equiv.), MeCN/H_2_O 19 : 1, 80 °C, 1.5 h; (ii) AcCl (1.15 equiv.), Py (1.25 equiv.), DCM, 0 °C, 5 min; then RT, 1 h; (iii) LiHMDS (1.8 equiv.), THF, MW (100 °C, 30 min); (iv) Boc_2_O (2.2 equiv.), DMAP (2.5 equiv.), THF, RT, 24 h; (v) TBAF (1 equiv.), ethylenediamine (2 equiv.), THF, 0 °C, 15 min; (vi) NaH (1.35 equiv.), MeCN, 0 °C, 3 h; (vii) **7** (1.2 equiv.), 0 °C, 10 min; then RT, 2 h; (viii) NaOMe (6 equiv.), MeCN, 50 °C, 20 min; (ix) DMTr-Cl (1.3 equiv.), Py, 0 °C, 5 min; then RT, 1.5 h; (x) chloro-(2-cyanoethoxy)diisopropylaminophosphine (2 equiv.), *N*-methylmorpholine (4 equiv.), CH_2_Cl_2_, RT, 2 h.

The method developed for the preparation of qAN1 and qA_nitro_ was adopted for the synthesis of pA (**1**).^11^ The DMTr-protected phosphoramidite of pA (**2**, Scheme 1) was synthesized over 9 steps with an overall yield of 16%, starting from the substituted deazapurine **3**. Suzuki–Miyaura cross-coupling of compound **3** with 3-amino-2-iodo-naphtalene (**6**) furnished **3a**, which was subjected to acetylation to activate the amine for nucleophilic aromatic substitution to yield **3b**. LiHMDS-mediated intramolecular cyclization of **3b** provided compound **4** in high yield. Subsequent Boc protection of the secondary amine, followed by removal of the *t*-butyldimethylsilyloxymethyl (TBDMSOM) protecting group using TBAF and ethylenediamine gave **5**. Compound **5** was *N*-glycosylated using Hoffer's α-chloro-sugar (**7**),^30^ and after global deprotection, the pA monomer **1** was obtained in excellent yield. Subsequent DMTr-protection of the primary alcohol and phosphitylation of the secondary alcohol of **1** furnished the phosphoramidite building block **2**.

### Photophysical properties of the pA nucleoside

**Fig. 1:**
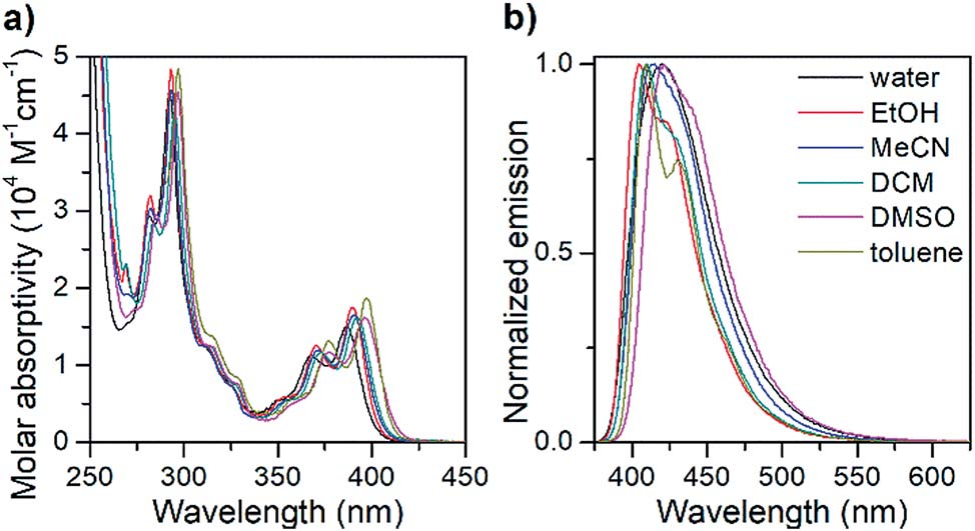
Isotropic absorption spectra (a) and normalized fluorescence emission spectra (b) of the pA nucleoside monomer (**1**) in various solvents.

The photophysical properties of the pA nucleoside (**1**) were determined in a variety of protic and aprotic solvents, covering a wide polarity range (Table 2 and Fig. 1).

**Table 2:** Photophysical properties of the pA nucleoside monomer (**1**) in different solvents

Solvent	*λ*_Abs,1_ (nm)	*ε*_1_ (M^–1^ cm^–1^)	*λ*_Abs,2_ (nm)	*ε*_2_ (M^–1^ cm^–1^)	*λ*_Em_ (nm)	*Φ*_F_ (%)	*ε*_1_ *Φ*_F_ ^*a*^ (M^–1^ cm^–1^)
Water^*b*^	387	15 200	368	11 400	420	66	10 100
DMSO	396	17 100	378	12 500	421	84	14 400
EtOH	390	17 500	370	12 600	405	66	11 600
DCM	392	16 100	373	11 700	410	74	11 900
Toluene	397	18 700	377	13 200	409	72	13 400
MeCN	390	16 500	372	11 900	414	64	10 500

^*a*^Brightness is calculated as the product of the fluorescence quantum yield (*Φ*_F_) and the molar absorptivity at the long-wavelength maximum (*ε*_1_).

^*b*^With 2% DMSO.

The absorption spectrum of the pA nucleoside in water is characterized by two peaks at 368 and 387 nm, with molar absorptivities of 11 400 and 15 200 M^–1^ cm^–1^, respectively, as well as a strong absorption peak at 292 nm. In less polar solvents, these absorption peaks are slightly sharper with a red-shift of up to 10 nm. The emission spectrum of pA in water shows a single emission peak with a maximum at 420 nm (Fig. 1), whereas in less polar solvents a 20 nm red-shifted shoulder is visible. The largest Stokes shift, 33 nm (corresponding to 2000 cm^–1^), is observed in water.

Importantly, the pA nucleoside (**1**) retains an excellent fluorescence quantum yield across all solvents investigated (64% ≤ *Φ*_F_ ≤ 84%, Table 2), resulting in brightness values of above 10 000 M^–1^ cm^–1^ in all solvents, and making it the brightest base analogue monomer reported to date (see Table 1). The quantum yield in water (66%) is comparable to that of the widely used 2-aminopurine (68%),^16^ and is significantly higher than that of a majority of other bright FBAs found in the literature (for details see Table 1).

### Incorporation of pA into DNA oligonucleotides

To study the effect of replacing adenine with the size-expanded pA on DNA structure and stability, as well as the sensitivity of the photophysical properties of pA to neighbouring bases, we synthesized 16 different pA-modified DNA decamer sequences (Table 3). These oligonucleotides cover all possible combinations of neighbouring bases to pA. Stepwise coupling efficiencies were determined by automated trityl cation conductivity monitoring and exceeded 98% for all oligonucleotides synthesized, including oligonucleotides containing pA. For details of the solid-phase oligonucleotide synthesis, see the ESI.†

**Table 3:** Melting temperatures of pA-modified duplexes (*T*pAm), unmodified duplexes (*T*Am), and the difference (Δ*T*_m_) between them

NN^*a*^	Sequence	*T* pA m (°C)	*T* A m (°C)	Δ*T*_m_ (°C)
AA	5′-d(CGCA**A**(pA)**A**TCG)-3′	40.8	43.5	–2.7
AC	5′-d(CGCA**A**(pA)**C**TCG)-3′	45.3	47.1	–1.8
AG	5′-d(CGCA**A**(pA)**G**TCG)-3′	42.4	45.9	–3.5
AT	5′-d(CGCA**A**(pA)**T**TCG)-3′	44.8	43.4	1.4
CA	5′-d(CGCA**C**(pA)**A**TCG)-3′	49.9	46.5	3.4
CC	5′-d(CGCA**C**(pA)**C**TCG)-3′	54.6	50.3	4.3
CG	5′-d(CGCA**C**(pA)**G**TCG)-3′	52.2	49.5	2.7
CT	5′-d(CGCA**C**(pA)**T**TCG)-3′	53.2	47.3	5.9
GA	5′-d(CGCA**G**(pA)**A**TCG)-3′	43.0	45.3	–2.3
GC	5′-d(CGCA**G**(pA)**C**TCG)-3′	48.4	49.2	–0.8
GG	5′-d(CGCA**G**(pA)**G**TCG)-3′	47.6	48.1	–0.5
GT	5′-d(CGCA**G**(pA)**T**TCG)-3′	46.4	45.4	1.0
TA	5′-d(CGCA**T**(pA)**A**TCG)-3′	43.0	41.1	1.9
TC	5′-d(CGCA**T**(pA)**C**TCG)-3′	46.1	43.7	2.4
TG	5′-d(CGCA**T**(pA)**G**TCG)-3′	45.3	43.6	1.7
TT	5′-d(CGCA**T**(pA)**T**TCG)-3′	45.8	40.6	5.2

^*a*^Sequences are named by the bases neighbouring pA on the 5′- and 3′-sides, respectively. Unmodified samples contain an adenine instead of pA. Duplexes were formed by hybridization with the complementary strand as described in the experimental section (see ESI). The melting temperatures were calculated as the maximum of the first derivative of the UV-melting curves with a standard error of ≤0.3 °C. For individual error values, see Table S1.

### Conformation and stability of pA-modified duplexes

Circular dichroism (CD) analysis of the 16 strands annealed with their complementary strands shows the archetypal characteristics of B-form DNA, a positive band between 260 and 280 nm and a negative band around 245 nm (Fig. S1 and S2†), indicating that duplexes modified with pA adopt normal B-form geometry.^31^ There are minor differences between CD-spectra of the modified and unmodified duplexes, which most likely stem from differences in the absorption spectra of pA and adenine. In a similar manner to the quadracyclic adenine analogues qA and qAN1, the long-wavelength absorption band of pA was not observed in any of the CD-spectra.^11^,^32^

To investigate the effect of pA-incorporation on the stability of DNA duplexes, the melting temperatures of all pA-modified and unmodified duplexes were measured (Table 3). The UV-melting curves of all pA-modified duplexes (data not shown) have the general shape of the corresponding unmodified duplex, strongly indicating that normal B-form DNA is formed when adenine is replaced with pA. On average, pA-incorporation increases the DNA duplex melting temperature, *T*_m_, by 1.1 °C, a significantly smaller change than that of the quadracyclic adenine analogs qA (3.0 °C)^32^ and qAN1 (2.9 °C).^11^ Overall, pA with a 5′-purine neighbour has a destabilizing effect, while a 5′-pyrimidine or a 3′-thymine neighbour has a stabilizing effect. This observation is in line with the results for both qA and qAN1, which have been attributed to the larger increase in base-stacking overlap that occurs between the extended ring system of qA/qAN1 and a 5′-pyrimidine compared to a 5′-purine.^11^,^32^

Due to the correlation between thermal stability of the DNA duplex and neighbouring bases, the user can fine-tune the relative melting temperatures of pA-modified duplexes as compared to their unmodified counterparts. The small overall increase in melting temperature of pA–DNA is generally preferable over other adenine FBAs such as 2-AP,^33^ 3-MI,^28^ 6MAP, DMAP,^34^ and xA,^35^ which reduce the duplex stability.

**Fig. 2:**
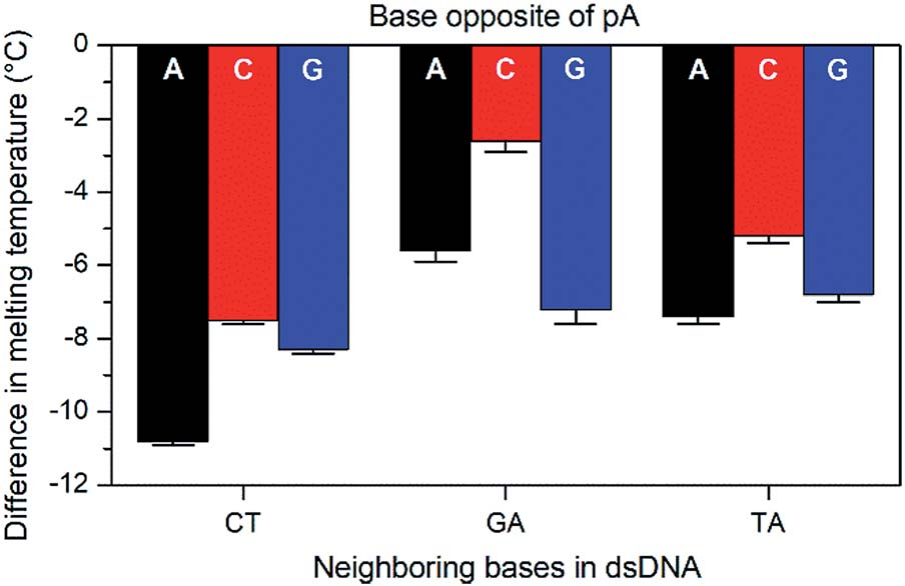
Difference in *T*_m_ with standard error between mismatched (A, C, or G opposite pA) and the corresponding matched sequences (T opposite pA) for three sets of pA nearest neighbours (CT, GA, and TA).

The base-pairing specificity of pA was evaluated by the change in melting temperature upon annealing three sequences (CT, GA and TA, see Table 3) with complementary sequences containing mismatched adenine, cytosine or guanine opposite pA (Fig. 2 and Table S2†).

The sequences were chosen to investigate the influence of neighbouring pyrimidines (CT), purines (GA) or one of each (TA). The melting temperature decreases by 2.6 to 10.8 °C when a base other than thymine is opposite pA, indicating that pA is selective for thymine. Unlike the parent compound qA and qAN1, where the smallest decrease was observed for guanine and adenine mismatches, respectively (qA: avg. for G: 9.8 °C *vs.* 15.3 °C for A/C; qAN1: avg. for A: 6.3 °C *vs.* 10.2 °C for C/G),^11^,^32^ the decrease is smallest when pA is opposite a cytosine and between two purines (GA). A possible explanation for this is that the extended aromatic structure of pA makes it easier to stack efficiently with its neighbouring bases while accommodating the mismatch. In that case, the bicyclic purines would offer a more efficient stacking than monocyclic pyrimidine neighbours, while the cytosine mismatch may be more easily accommodated than the larger purines.

### Photophysical properties of pA in ssDNA and dsDNA

**Fig. 3:**
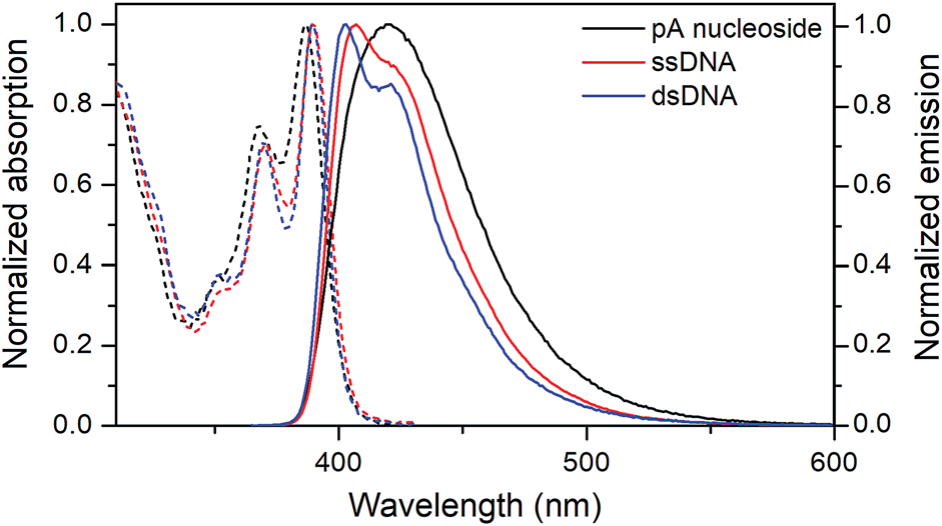
Normalized absorption (dashed) and emission (solid) spectra of the pA nucleoside (**1**, black), and of the pA-containing sequence AA (Table 3) as single- (red) and double-stranded (blue) DNA.

The absorption and emission properties of pA in single- and double-stranded DNA were measured for all 16 combinations of neighbouring bases (Table 4 and Fig. S3†). Fig. 3 shows representative absorption and emission spectra of pA in ssDNA and dsDNA. The absorption maximum of pA is slightly red-shifted when incorporated into DNA, and, importantly for its utility, lies well outside the absorption range of the natural nucleobases (390 ± 2 nm for all sequences in single- and double-stranded DNA), allowing selective excitation.^36^ The emission peak of pA is blue-shifted upon incorporation in DNA to around 407 nm for ssDNA and 403 nm for dsDNA, compared to 420 nm for the monomer, but still lies in the visible region. This blue-shift is typical for FBAs inside nucleic acids, and is due to the less polar environment inside DNA which leads to reduced solvent relaxation.^37^

**Table 4:** Photophysical properties of single- and double-stranded DNA sequences containing pA

NN^*a*^	Single strands				Double strands			
	*ε*_Abs_ ^*b*^ (M^–1^ cm^–1^)	*λ*_Em_ (nm)	*Φ*_F_ ^*c*^ (%)	*εΦ*_F_ (M^–1^ cm^–1^)	*ε*_Abs_ ^*b*^ (M^–1^ cm^–1^)	*λ*_Em_ (nm)	*Φ*_F_ ^*c*^ (%)	*εΦ*_F_ (M^–1^ cm^–1^)
AA	14 500	407	58	8400	13 800	403	22	3000
AC	15 000	406	11	1700	14 300	402	6.2	890
AG	15 200	405	24	3600	13 200	403	16	2100
AT	15 000	407	15	2300	14 500	400	17	2500
CA	14 200	408	14	2000	12 600	405	9.9	1200
CC	14 500	407	3.6	520	12 900	406	4.3	560
CG	14 700	405	6.1	900	12 400	406	8.2	1000
CT	14 000	409	4.1	570	13 500	403	10	1400
GA	15 000	407	42	6300	14 000	404	14	2000
GC	16 000	404	4.4	700	14 700	405	3.4	500
GG	14 300	406	28	4000	13 200	405	13	1700
GT	15 200	407	8.3	1300	14 400	401	7.7	1100
TA	14 100	408	6.2	870	13 300	402	8.0	1100
TC	14 400	406	2.4	350	13 000	403	4.4	570
TG	14 500	408	3.3	480	12 600	404	6.7	840
TT	14 100	409	2.6	370	13 600	400	11	1500

^*a*^Sequences are named according to nucleotides surrounding pA; full sequences can be found in Table 3.

^*b*^Molar absorptivity values are reported as an average of two or more experiments with a standard error of ≤200.

^*c*^Quantum yields were determined for the emission profiles shown in Fig. S3, with quinine sulfate as reference (*Φ*_F_ = 54.6% in 0.5 M H_2_SO_4_), using an excitation wavelength of 353 nm, and are reported as an average of two or more experiments with a standard error of ≤0.8%.

Both the absorption and emission are more structured inside DNA, indicating that pA is firmly stacked and protected from aqueous solvation. One additional advantage of pA over qAN1 in future applications is that unlike qAN1, none of the pA duplexes have an additional shoulder in their emission spectrum (Fig. S3†), indicating that for pA, no significant tautomerization occurs in the excited state.^11^

The quantum yield of pA inside dsDNA is significantly higher than that of qAN1, reported to be one of the brightest adenine FBAs in DNA (Tables 1 and 4).^11^ On average, the fluorescence quantum yield of pA is 15% in ssDNA and 10% in dsDNA (Table 4, Fig. S4†), resulting in average brightness (*ε* × *Φ*_F_) values of 2130 M^–1^ cm^–1^ and 1370 M^–1^ cm^–1^, respectively. This represents a 2.7-fold increase in average brightness in dsDNA compared with qAN1 and ranks pA top three among characterized FBAs (Table 1; also note that 6-MI has been studied for only a few sequences).

The quantum yield of pA is dependent on the neighbouring bases (Table 4, Fig. S4†), and is lowest when pA is flanked by pyrimidines in ssDNA or by a 5′-cytosine in dsDNA, the latter being the only instance where qAN1 has a higher quantum yield than pA (Fig. S5†).

Apart from sequence GT, all sequences with a neighbouring thymine show a higher quantum yield in dsDNA than in ssDNA. The same trend was noted for qAN1, and was attributed to reduced stacking interaction between thymine and qAN1 in the helical duplex structure.^11^ The quantum yield is highest when pA is flanked by purines, especially adenine which yields unprecedented FBA brightness values (for comparison see Table 4) of 8400 M^–1^ cm^–1^ and 3000 M^–1^ cm^–1^ in ssDNA (*Φ*_F_ = 58%) and dsDNA (*Φ*_F_ = 22%), respectively, which is even higher than the top values for tC^O^ (having the highest average brightness, Table 1). Inside DNA, the decrease in quantum yield of pA is accompanied by a shortening of its fluorescence lifetime, with amplitude-weighted mean lifetimes in dsDNA ranging from 3.4 ns for sequence AA to 0.7 ns for GC (Table S3;† detailed lifetime analysis manuscript is in preparation).

### pA in interbase FRET

**Fig. 4:**
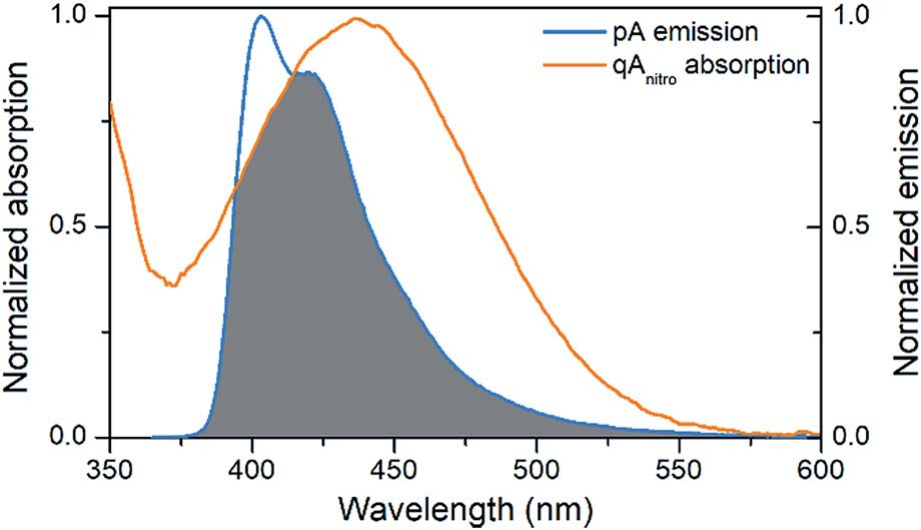
Visualization of the spectral overlap between pA emission and qA_nitro_ absorption in dsDNA. Spectra are normalized at their long-wavelength maxima.

The emission of pA overlaps well with the absorption of the FRET-acceptor qA_nitro_ (Fig. 4),^11^ with a calculated Förster radius, *R*_0_, of 21–28 Å (average 24.4 Å), indicating that the FRET pair could be used to monitor distances slightly beyond 1.5 turns of the B-DNA helix.^38^ In the *R*_0_ calculations, the orientation factor, *κ*^2^, was set to 2/3 for comparative purposes, but for firmly stacked probes in DNA, the value of *κ*^2^ is expected to vary with the relative orientation of the donor and acceptor, which depends on the number of bases separating them.^11^,^38^

**Fig. 5:**
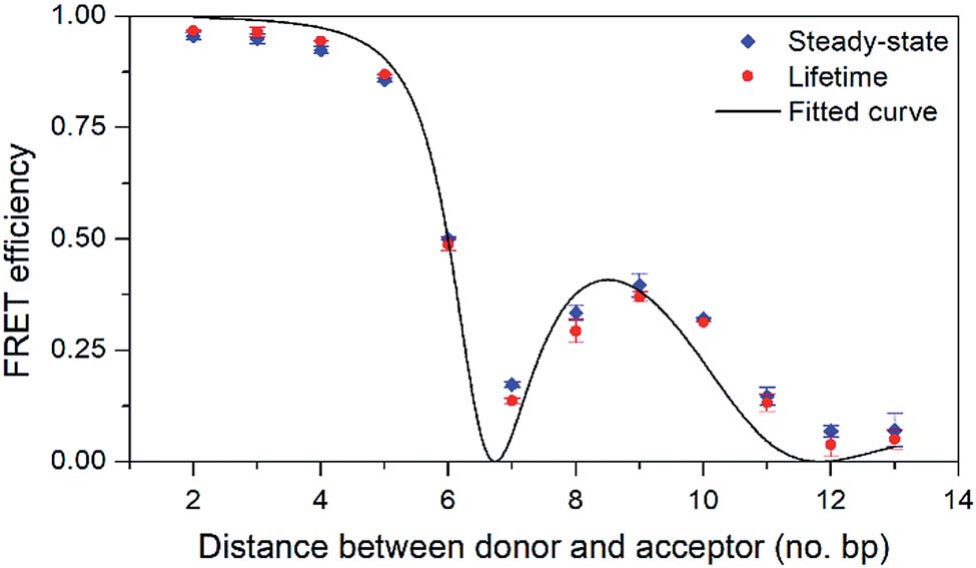
FRET efficiency with 95% confidence intervals as a function of the number of base pairs separating the donor (pA) and acceptor (qA_nitro_). Diamonds and circles mark data from steady-state and lifetime measurements, respectively. The line shows the curve fitted to the data based on FRET-theory (eqn (S9)†).

To evaluate the FRET efficiency of the pA–qA_nitro_ pair at various donor–acceptor distances, eight 33-mer sequences were prepared (Table 5): three donor strands containing pA (all with adenine neighbours), four complementary acceptor strands containing qA_nitro_, and one unmodified complementary strand. First, the quantum yields of pA at the three donor positions were measured in duplexes without acceptor. The quantum yield was found to depend on position, increasing (from 20 to 28%) as pA is positioned further from the 5′-end. A similar trend was observed for qAN1 in the same sequences, and may be due to changes in the local environment at each position. The FRET efficiency of each 12 combinations of the donor and acceptor sequences, corresponding to a separation of 2–13 base-pairs, was determined using both the decrease in steady-state emission and the shortening of the average lifetime of pA (see Fig. 5 and Table S4†).

**Table 5:** Synthesized 33-mer sequences used for interbase FRET measurements

Sequence name^*a*^	DNA sequence^*b*^	*Φ*_F_ ^*c*^ (%)
D7	5′-d(CGA TCA (**pA**)AA AAA ATT **W**)-3′	20
D9	5′-d(CGA TCA AA(**pA**) AAA ATT **W**)-3′	24
D11	5′-d(CGA TCA AAA A(**pA**)A ATT **W**)-3′	28
A0	5′-d(**X** TAT AAT CGT AAT TTT **Z**)-3′	
A13	5′-d(**X** TAT **qA**_**nitro**_ AT CGT AAT TTT **Z**)-3′	
A14	5′-d(**X** TAT A **qA**_**nitro**_ T CGT AAT TTT **Z**)-3′	
A19	5′-d(**X** TAT AAT CGT **qA**_**nitro**_ AT TTT **Z**)-3′	
A20	5′-d(**X** TAT AAT CGT A **qA**_**nitro**_ T TTT **Z**)-3′	

^*a*^Sequence and sample preparation can be found in the ESI. Samples are named by the donor (D) or acceptor (A) position from the 5′ end.

^*b*^ **W** = ACG ATT ATA AGG AGG AGG. **X** = CCT CCT CCT; **Z** = TTT TGA TCG.

^*c*^Quantum yields were determined with quinine sulfate as reference (*Φ*_F_ = 54.6% in 0.5 M H_2_SO_4_) using an excitation wavelength of 353 nm.

As can be seen in Fig. 5, the FRET efficiency is high at short distances, but varies periodically as the donor–acceptor distance increases, which indicates that pA and qA_nitro_ are firmly stacked inside DNA. An in-house designed MATLAB script was used to fit a function based on FRET-theory to the data (see eqn (S9)†). The optimal fit is obtained for an overlap integral of 1.8 × 10^14^ M^–1^ cm^–1^ nm,^4^ and a phase angle of 43° (the angle between the donor and acceptor transition dipole moment projected onto the base-pair plane, when donor and acceptor are in adjacent base pairs).^38^ Using the spectral profiles of pA and qA_nitro_, the overlap integral was calculated to be 1.5 × 10^14^ M^–1^ cm^–1^ nm,^4^ which is indeed close to the fitted value. Using time-dependent density functional theory (TDDFT)-calculations, the orientation of the transition dipole moments of pA and qA_nitro_ have been predicted, and suggests an associated phase angle of 43° in B-form DNA, which is also in good agreement with the fitted value (experimental determination of the transition dipole moments of pA and qA_nitro_ is the subject of a separate study; manuscript in preparation). In summary, pA is an excellent FRET donor that surpasses qAN1 in brightness and allows for base–base FRET-studies at longer distances than qAN1.

### pA as a two-photon chromophore

**Fig. 6:**
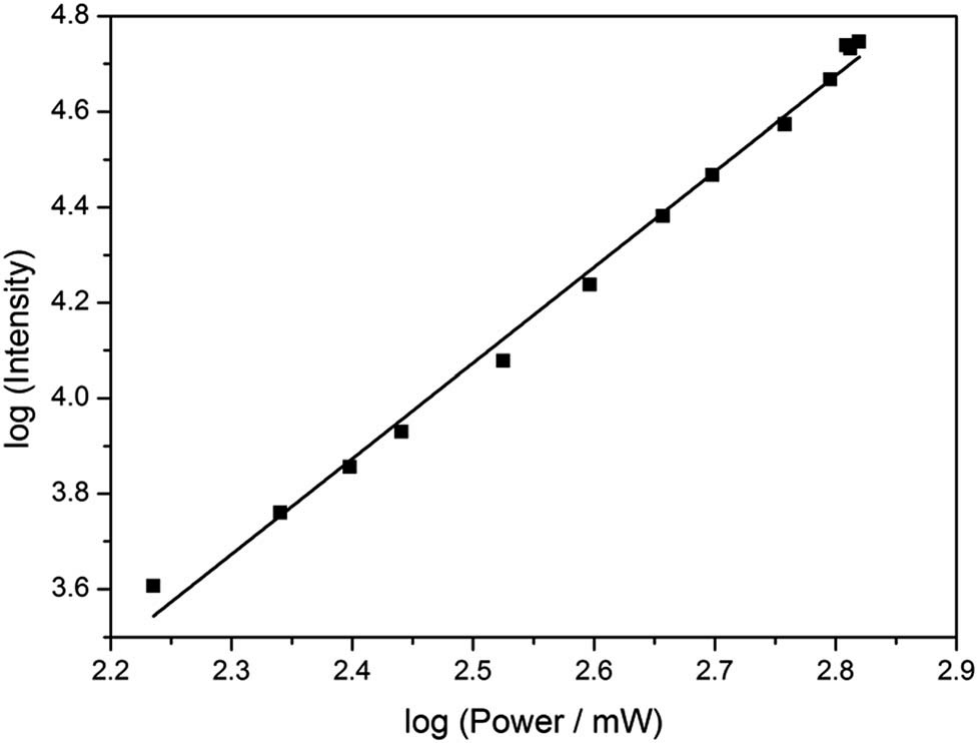
Plot of log (fluorescence intensity) *vs.* log (laser power) for pA in EtOH, showing a square-law dependence. The excitation wavelength was 780 nm. The errors were calculated to <5% from the standard deviation of three emission spectra collected at each power.

Based on its exceptionally high one-photon brightness for an FBA, we anticipated that pA might have a potential as two-photon probe. Two-photon excitation at 780 nm was verified by measuring the fluorescence intensity as a function of laser power (Fig. 6). The log–log plot shows a slope of 2.00 (±0.05), confirming a two-photon process. The two-photon cross-section of the pA base at 780 nm was measured to be 6.6 GM (Table S5†), which combined with its high quantum yield makes the two-photon brightness, *Φ*_F_*σ*^2^ = 5.3 GM, significantly higher than 5-(thiophen-2-yl)-6-aza-uridine, TPAU (*Φ*_F_*σ*^2^ = 0.76 GM for 690 nm excitation, Table 1; Chart S1† for structure), and the pteridines 6-MI and 6MAP (*Φ*_F_*σ*^2^ = 1.75 and 1.33 GM, respectively, Table 1; Chart 1 for structure), which are the brightest two-photon excitable FBAs reported to date (Table 1).^27^ Importantly, the excitation wavelength of 780 nm for pA is also ideal for conventional Ti–sapphire laser excitation. When incorporated into an oligonucleotide (Table 3, sequence GA), the two-photon cross section was measured as 3.0 GM in ssDNA and 2.4 GM in dsDNA of this sequence, yielding highly promising FBA two-photon brightness values of 1.3 GM and 0.35 GM in ssDNA and dsDNA, respectively (Tables S6 and S7†).

The two-photon brightness of pA is significantly higher than that of other FBAs (Table 1), in general more than an order of magnitude. Moreover, pA keeps a high two-photon cross section inside DNA and is similar to that of coumarine-120 (cross section of 3 GM in water), which has been detected previously at the single-molecule level.^47^ We therefore hypothesize that pA may become the first practical single-molecule FBA probe. Overall, the two-photon properties of pA as a monomer and inside DNA are unmatched by previous FBAs and, in combination with its excellent one-photon emissive properties, this makes pA is a highly versatile fluorophore for biological purposes.

### pA in single-particle microscopy

**Fig. 7:**
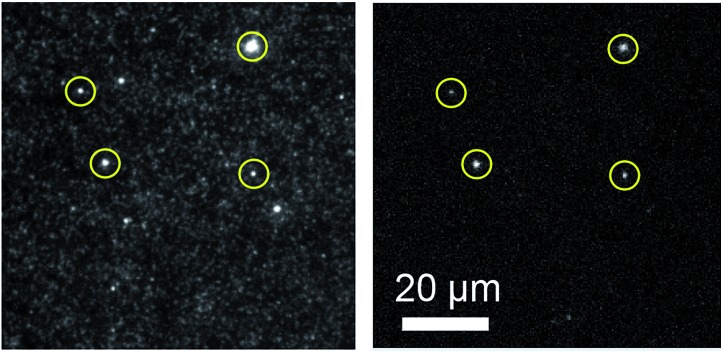
TIRF images of liposome systems labelled with Rhodamine B-conjugated lipids and cholesterol-anchored pA-modified dsDNA. Left: visualization by excitation of Rhodamine B using a TRITC filter. Right: visualization of the same liposomes by exciting pA using a DAPI filter.

The superior brightness of pA compared with other FBAs opens possibilities for its use as a fluorescent label in microscopy applications. To test this we used the general features of a liposome–DNA construct already established in-house.^48^ In this preliminary study, liposomes decorated with double cholesterol-anchored dsDNA binding five AA pA-sequences (Table 3; adenine-flanked pA) through a 50-mer overhang containing 5 × 10-mer repeats of the complementary sequence, were observed using TIRF microscopy (Fig. 7; for details of sample preparation, sequences of the anchoring cholesterol DNAs, and microscopy setup see ESI†). Even though adenine-flanked pA is approximately 24 times less bright than the membrane-attached Rhodamine B (*ε* × *Φ*_F_ = 68 900 M^–1^ cm^–1^), and at most 2.5 times more abundant than rhodamine in the liposome constructs (details in ESI†), it is still possible to visualize the same liposome systems by exciting pA (circles in Fig. 7, left and right). This serves as an example that pA indeed can be used for visualization purposes in microscopy studies at concentrations comparable to those of commercially available fluorophores. In contrast to FBAs, such fluorophores are commonly used to externally label DNA using covalent linkers at the end of the sequences. Our proof-of-concept experiment shows the possibility of using FBAs in microscopy-based investigations of nucleic acids, for example, monitoring and tracking of DNA/RNA in live cells. Compared to the external labels our novel FBA offers some important advantages. For example, pA can be placed directly into the duplex close to a site of interest without perturbing the DNA structure, where it can serve as a sensitive and spatially fixed probe. Moreover, external probes with their combined hydrophobic and charged properties are prone to interact with lipids of liposomal, lipid nanoparticle (LNP) and cell membrane systems, whereas this is considerably less likely for pA, and other FBAs, which are hidden away in the interior of the nucleic acid sequence. We suggest that this will be advantageous in, for example, microscopy investigations of uptake, trafficking, delivery and release of nucleic acid-based drug candidates formulated in lipid-based systems.

## Conclusions

Fluorescent base analogues offer important advantages over extrinsic probes, but due to the current slight shortcomings in photophysical properties they have yet to achieve the wide applicability of conventional linker-coupled labels. In this work, we report a highly fluorescent adenine analogue, pA, that has exceptional one- and two-photon brightness as a monomer and, importantly, when incorporated into DNA. Despite its size, we find that pA seems to only minimally perturb B-form DNA and keep the sequence specificity of normal A. The photophysical properties of pA compare favourably with, or outperform, all previously reported fluorescent base analogues (Table 1). The versatility of pA is demonstrated through its use as a FRET donor, as a two-photon excitation probe, and as a bright label in microscopy, and we foresee its broad application. Furthermore, we hypothesize that pA may become the first practical single-molecule observable FBA using two-photon excitation methodology. We are currently exploring the possibility of using pA in single-molecule experiments to facilitate investigations of the structure and dynamics of oligonucleotides as well as their interactions with, for example, small molecules, peptides and proteins.

## Conflicts of interest

There are no conflicts to declare.

## Supplementary Material

Supplementary information
