# Supplementary material for: Pentacyclic adenine: a versatile and exceptionally bright fluorescent DNA base analogue
Source: Chem Sci. 2018 Mar 1;9(14):3494–502. doi: 10.1039/c7sc05448c (PMC5934695; doi:10.1039/c7sc05448c)
Supplement: Supplementary file 1 [file SC-009-C7SC05448C-s001.pdf]

Electronic Supplementary Material (ESI) for Chemical Science.
This journal is © The Royal Society of Chemistry 2018

Supporting Information for:

Pentacyclic adenine: a versatile and exceptionally bright fluorescent DNA base analog

Mattias Bood, Anders F. Füchtbauer, Moa S. Wranne, Jong Jin Ro, Sangamesh Sarangamath, Afaf H. El-Sagheer, Déborah L. M. Rupert, Rachel S. Fisher, Steven W. Magennis, Anita C. Jones, Fredrik Höök, Tom Brown, Byeang Hyean Kim, Anders Dahlén, L. Marcus Wilhelmsson* and Morten Grøtli**

Table of Contents

1. Synthesis and characterization of pA....2
- 1.1. General Experimental Details....2
- 1.2. Synthesis and Characterization....3
- 1.3. Spectral data....6

2. Oligonucleotide Synthesis and Analysis....14
- 2.1 Oligonucleotide Synthesis....14
- 2.2. Oligonucleotide Analytical Data....15

3. Photophysical Experimental Section....19
- 3.1. Molar absorptivity and quantum yield of the pA monomer....19
- 3.2. Hybridization of DNA-strands....19
- 3.3. DNA UV-melting and Circular Dichroism (CD)....19
- 3.4. Fluorescence measurements....19
- 3.5. FRET measurements....20
- 3.6. Data evaluation/curve fitting....20

4. TIRF experimental setup....21
- 4.1. Materials....21
- 4.2. Vesicle preparation....21
- 4.3. Size distribution and concentration of vesicles with Nanoparticle Tracking Analysis....22
- 4.4. Vesicle functionalization with pA-cholesterol-DNA complex....22
- 4.5. Total internal reflection fluorescence (TIRF) microscopy and image analysis....22
- 4.6. Sample holder and binding of vesicles to surface....22

5. Additional figures, tables, charts and notes....24
- 5.1. Chart S1....24
- 5.2. Chart S2....24
- 5.3. Figure S1....25
- 5.4. Figure S2....26
- 5.5. Table S1....27
- 5.6. Table S2....28
- 5.7. Figure S3....29
- 5.8. Figure S4....29
- 5.9. Figure S5....29
- 5.10. Table S3....30
- 5.11. Table S4....31

6. Two-Photon Excitation....32
- 6.1. Table S5....32
- 6.2. Table S6....33
- 6.3. Table S7....33

7. References....34

1. Synthesis and characterization of pA

1.1. General Experimental Details

All reactions were performed in oven-dried glassware under a nitrogen atmosphere, unless otherwise stated. Reagents were purchased from various chemical vendors and used as received without further purification. Solvents were purchased as HPLC grade and used as received. Microwave reactions were performed in a Biotage Initiator Reactor using single mode irradiation with temperature and pressure control and with fixed hold time on. Reactions were monitored by TLC (Merck silica gel 60 F_{254}) analyzed under UV (254 nm), and by UPLC-MS (ESI/UV), using a Waters Acquity system equipped with either an Acquity UPLC HSS C_{18} column (1.8 µm, length 50 mm, ID 2.1 mm) with a gradient of water/MeCN (95:5 to 5:95) , with the water eluent containing 1% formic acid (pH 3) or an Acquity UPLC BEH C_{18} column (1.7µm, length 50 mm, ID 2.1 mm) with a gradient of water/MeCN (95:5 to 5:95), with the water eluent containing 1% ammonium hydroxide (pH 10). Flash chromatography was performed on a Grace Reveleris X2 instrument using pre-packed silica columns. HPLC purification was performed with formic acid (pH 3) or ammonia (pH 10) as modifier on a preparative HPLC system with an XBridge C_{18} column (10 µm, 250 × 50 mm). ^{1}H and ^{13}C NMR spectra were recorded at 300 K on a Bruker 500 MHz system equipped with a CryoProbe, operating at 500 MHz and 126 MHz, respectively. The chemical shifts are recorded in ppm relative to the solvent residual peaks: $CDCl_3$ (7.26 ppm for ^{1}H and 77.15 ppm for ^{13}C) or DMSO-d_6 (2.50 ppm for ^{1}H and 39.52 ppm for ^{13}C). High resolution LC-MS was detected on a Waters LCTp ToF MS using electrospray ionization (ESI-MS). The MS inlet consisted of a Waters Acquity UPLC system, and the separation was performed on a Waters C18 XBridge at 45-50 °C. The separation was obtained with a 2-95% ACN gradient over 3 min at pH 10 (40 mM NH3 and 5 mM H2CO3). A measure of related impurities was assessed at 210 nM.

1.2. Synthesis and Characterization

3-[7-({[*tert*-Butyl(dimethyl)silyl]oxy}methyl)-4-chloro-7*H*-pyrrolo[2,3-*d*]pyrimidin-5-yl]naphthalen-2-amine (3a)

Compound **3** (8.84 g, 20.9 mmol, 1.1 equiv), 3-iodonaphtalen-2-amine (5.10 g, 19.0 mmol, 1.0 equiv) and potassium carbonate (6.55 g, 47.4 mmol, 2.5 equiv) was dissolved in MeCN (180 mL) and water (9.5 mL). Bis(triphenylphosphine)palladium(II) dichloride (0.59 g, 0.83 mmol, 0.044 equiv) was added in one portion, and the mixture was heated to 80 °C for 90 min. Then water (40 mL) was added to the mixture, which was stirred vigorously for 5 min, and then allowed to stand for 5 min without stirring, to allow the phases to separate. The organic phase was dried over Na_2SO_4, filtered, concentrated and purified by flash chromatography (SNAP 100 g; DCM in heptane: 10-100%) to afford **3a** (5.87 g, 71%) as a white solid.

^{1}H NMR (DMSO-d_6) δ ^{1}H NMR (500 MHz, DMSO) δ 8.72 (s, 1H), 7.87 (s, 1H), 7.67 (d, *J* = 8.0 Hz, 1H), 7.60 (s, 1H), 7.56 (d, *J* = 8.2 Hz, 1H), 7.31 (t, *J* = 7.5 Hz, 1H), 7.12 (t, *J* = 7.3, Hz, 1H), 7.00 (s, 1H), 5.89 (s, 2H), 5.00 (s, 2H), 0.86 (s, 9H), 0.13 (s, 6H). ^{13}C NMR (126 MHz, DMSO-d_6) δ 151.1, 150.9, 150.6, 146.0, 134.8, 130.7, 129.7, 127.5, 126.07, 126.03, 124.8, 121.21, 121.17, 115.9, 112.0, 106.3, 67.9, 25.6, 17.8, -5.2. HRMS (ESI-TOF) *m/z* calcd for $C_{23}H_{28}ClN_4OSi^+$ [M + H]$^+$: 439.1721, found: 439.1753.

2-({[*tert*-Butyl(dimethyl)silyl]oxy}methyl)-2,6-dihydro-2,3,5,6-tetraazacyclopenta[*de*]tetracene (4)

AcCl (1.10 mL, 15.5 mmol, 1.15 equiv) was added dropwise to a solution of **3a** (5.90 g, 13.4 mmol) in DCM (150 mL) and pyridine (1.36 mL, 16.8 mmol, 1.25 equiv) at 0 °C. After 5 min, the mixture was allowed to return to RT. After a total of 1 h, satd. aq. $NaHCO_3$ (70 mL) was added. The organic phase was isolated, dried (Na_2SO_4), and concentrated to afford a pale-yellow foam (6.46 g).

The material was split in ten portions and added to oven-dried MW vials with magnetic stir bars and sealed. THF (12 mL) was added by syringe to each vial, followed by LiHMDS (1M in THF; 3.0 mL, 3.0 mmol, 1.8 equiv). The vials were heated to 100 °C in a microwave reactor for 30 min each. The reaction mixtures were pooled, and the material was concentrated *in vacuo*. The resulting orange solid was suspended in water (200 mL) through sonication, filtered, washed with ice cold EtOH (3 × 40 mL), and dried under vacuum overnight to afford **4** (3.94 g, 73%) as a pale yellow solid.

^{1}H NMR (500 MHz, DMSO-d_6) δ 10.86 (s, 1H), 8.22 (s, 1H), 8.21 (s, 1H), 7.79 (d, *J* = 8.0 Hz, 1H), 7.72 (d, *J* = 7.9 Hz, 1H), 7.58 (s, 1H), 7.53 (s, 1H), 7.39 – 7.42 (m, 1H), 7.34 – 7.37 (m, 1H), 5.74 (s, 2H), 0.85 (s, 9H), 0.10 (s, 6H). ^{13}C NMR (126 MHz, DMSO) δ 155.5, 153.5, 147.0, 137.5, 132.6, 129.6, 127.2, 126.5, 126.3, 124.7, 122.6, 120.4, 114.5, 113.2, 110.6, 106.0, 67.8, 25.6, 17.8, -5.2. HRMS (ESI-TOF) *m/z* calcd for $C_{23}H_{27}N_4OSi^+$ [M + H]$^+$: 403.1954, found: 403.1945.

***tert*-Butyl 2-({[*tert*-butyl(dimethyl)silyl]oxy}methyl)-2,3,5,6-tetraazacyclopenta[*de*]tetracene-6(2*H*)-carboxylate (4a)**

A 250 mL RB flask with magnetic stir bar was charged with **4** (3.94 g, 9.79 mmol), DMAP (2.99 g, 24.5 mmol, 2.5 equiv), sealed with a septum, evacuated and regassed with N_2 twice. THF (50 mL) and Boc_2O (4.95 mL, 21.5 mmol, 2.2 equiv) was added by syringe, and the vial was stirred at RT for 24 h. The mixture was concentrated and purified by flash chromatography (EtOAc in heptane: 15-40%) to afford **4a** (3.92 g, 80%) as a white solid.

^{1}H NMR (500 MHz, $CDCl_3$) δ 8.50 (s, 1H), 8.03 (s, 1H), 7.73 – 7.79 (m, 1H), 7.66 – 7.73 (m, 2H), 7.37 – 7.47 (m, 2H), 7.31 (s, 1H), 5.80 (s, 2H), 1.75 (s, 9H), 0.90 (s, 9H), 0.11 (s, 6H). ^{13}C NMR (126 MHz, $CDCl_3$) δ 155.2, 152.5, 151.2, 148.1, 135.6, 132.8, 130.5, 127.7, 127.2, 126.6, 125.9, 122.9, 120.7, 115.1, 114.2, 110.7, 106.8, 85.9, 68.5, 28.0, 25.8, 18.2, -5.0. HRMS (ESI-TOF) *m/z* calcd for $C_{28}H_{35}N_4O_3Si^+$ [M + H]$^+$: 503.2478, found: 503.2466.

***tert*-Butyl 2,3,5,6-tetraazacyclopenta[*de*]tetracene-6(2*H*)-carboxylate (5)**

A 250 mL RB flask with magnetic stir bar was charged with **4a** (3.92 g, 7.80 mmol) and THF (78 mL). The flask was sealed with a septum, and cooled to 0 °C in an ice bath. Ethylenediamine (1.05 mL, 15.6 mmol, 2.0 equiv) was added, followed by TBAF (1M in THF; 7.8 mL, 7.8 mmol, 1.0 equiv). After 15 min, satd. aq. NH_4Cl (20 mL) and water (100 mL) was added. The biphasic mixture was concentrated on a rotary evaporator (at 20 °C) until it was a clear, colorless solution with a white precipitate. The suspension was filtered, and the filtered solids were washed with water (3 × 75 mL) and Et_2O (3 × 40 mL), to afford **5** (2.57 g, 92%) as a white solid after drying under vacuum.

^{1}H NMR (500 MHz, DMSO-d_6) δ 12.15 (s, 1H), 8.32 (s, 1H), 8.29 (s, 1H), 7.80 – 7.87 (m, 2H), 7.66 (d, *J* = 1.1 Hz, 1H), 7.55 (s, 1H), 7.42 – 7.47 (m, 2H), 1.68 (s, 9H). ^{13}C NMR (126 MHz, DMSO-d_6) δ 154.4, 151.6, 150.6, 148.5, 135.1, 132.0, 130.0, 127.5, 126.9, 126.6, 125.9, 122.7, 120.8, 114.2, 113.2, 108.3, 105.5, 85.7, 27.4. HRMS (ESI-TOF) *m/z* calcd for $C_{21}H_{19}N_4O_2^+$ [M + H]$^+$: 359.1508, found: 359.1509.

***tert*-Butyl 2-(2′-deoxy-3′-*O*-[4-methylbenzoyl]-5′-*O*-[4-methylbenzoyl]-β-D-ribofuranosyl)-2,3,5,6-tetraazacyclopenta[*de*]tetracene-6(*2H*)-carboxylate (5a)**

A 250 mL RB flask with magnetic stir bar was charged with **5** (2.57 g, 7.16 mmol) and NaH (60% dispersion in mineral oil; 0.34 g, 8.6 mmol, 1.2 equiv). The flask was sealed with a septum, and was evacuated and regassed with N_2 twice. The flask was placed in an ice bath at 0 °C and MeCN (143 mL) was added by syringe and the flask sonicated for 30 s to disperse the NaH. After 2 h of stirring at 0 °C, additional NaH (43 mg, 1.1 mmol, 0.15 equiv) was added. After a total of 3 h, (2*R*,3*S*,5*R*)-5-chloro-2-(((4-methylbenzoyl)oxy)methyl)tetrahydrofuran-3-yl 4-methylbenzoate (**6**, Hoffer's α-chlorosugar; 3.34 g, 8.59 mmol, 1.2 equiv) was added in one portion, and the flask was sealed with a septum. After 10 minutes, the RM was returned to RT (color change to yellow over 30 min). After 2 h, the mixture was concentrated and purified by flash chromatography (EtOAc in DCM: 0-10%) to afford **5a** (2.90 g, 57%) as an off-white solid.

^{1}H NMR (500 MHz, $CDCl_3$) δ 8.49 (s, 1H), 8.01 (dd, *J* = 11.5, 8.2 Hz, 4H), 7.67 – 7.72 (m, 2H), 7.59 – 7.64 (m, 1H), 7.56 (s, 1H), 7.38 – 7.45 (m, 2H), 7.33 (s, 1H), 7.26 – 7.32 (m, 4H), 6.79 (dd, *J* = 8.1, 6.0 Hz, 1H), 5.83 (dt, *J* = 6.2, 2.1 Hz, 1H), 4.84 (dd, *J* = 11.7, 3.2 Hz, 1H), 4.62 – 4.68 (m, 2H), 3.01 (ddd, *J* = 14.5, 8.3, 6.4 Hz, 1H), 2.84 (ddd, *J* = 14.2, 5.8, 2.0 Hz, 1H), 2.45 (s, 3H), 2.37 (s, 3H), 1.73 (s, 9H). ^{13}C NMR (126 MHz, $CDCl_3$) δ 166.34, 166.25, 155.2, 152.5, 151.0, 144.6, 144.4, 135.4, 132.9, 130.4, 130.0, 129.9, 129.6, 129.4, 127.7, 127.09, 127.05, 126.7, 126.6, 125.9, 123.0, 120.3, 115.1, 111.7, 111.3, 107.2, 85.9, 84.9, 82.8, 75.5, 64.5, 39.0, 28.0, 21.91, 21.86. HRMS (ESI-TOF) *m/z* calcd for $C_{42}H_{39}N_4O_7^+$ [M + H]$^+$: 711.2819, found: 711.2807.

2-(2′-Deoxy-β-D-ribofuranosyl)-2,6-dihydro-2,3,5,6-tetraazacyclopenta[*de*]tetracene (1)

A 100 mL RB flask with magnetic stir bar was charged with **5a** (2.90 g, 4.08 mmol) and sealed with a cap. MeCN (27 mL) was added by syringe, followed by a dropwise addition of sodium methoxide (30% in MeOH; 4.66 mL, 24.5 mmol; 6 equiv). After heating to 50 °C for 20 min, the reaction was concentrated on the rotary evaporator (water bath: 40 °C). The resulting solid was dissolved in DMSO (50 mL), filtered and then purified by acidic RP-HPLC (25-60% MeCN) to afford **1** (1.51 g, 99%) as an off-white solid after lyophilization.

^{1}H NMR (500 MHz, DMSO-d_6) δ 10.88 (s, 1H), 8.18 (s, 1H), 8.15 (s, 1H), 7.79 (d, *J* = 8.0 Hz, 1H), 7.71 (d, *J* = 11.5 Hz, 2H), 7.54 (s, 1H), 7.40 (t, *J* = 7.1 Hz, 1H), 7.35 (t, *J* = 7.3 Hz, 1H), 6.43 (t, *J* = 6.9 Hz, 1H), 5.31 (s, 1H), 5.17 (s, 1H), 4.43 (s, 1H), 3.89 (s, 1H), 3.63 (s, 1H), 3.57 (s, 1H), 2.62 – 2.73 (m, 1H), 2.24 – 2.3 (m, 1H). ^{13}C NMR (126 MHz, DMSO-d_6) δ 155.2, 153.5, 146.9, 137.4, 132.6, 129.6, 127.2, 126.6, 126.3, 124.7, 122.4, 120.4, 113.3, 112.9, 110.4, 106.6, 87.7, 84.4, 71.2, 62.2, 39.5 (overlaps with DMSO). HRMS (ESI-TOF) *m/z* calcd for $C_{21}H_{19}N_4O_3^+$ [M + H]$^+$: 375.1457, found: 375.1439.

2-{2′-Deoxy-5′-*O*-[4,4'-dimethoxytrityl]-β-D-ribofuranosyl]-2,6-dihydro-2,3,5,6-tetraazacyclopenta[*de*]tetracene (1a)

A 25 mL RB flask was charged with **1** (0.800 g, 2.14 mmol), which was co-evaporated with pyridine (5 mL) two times. A magnetic stir bar was added, followed by 4,4'-dimethoxytrityl chloride (0.94 g, 2.8 mmol, 1.3 equiv) in one portion. The flask was sealed with a cap and evacuated and regassed with N_2 twice. The flask was then cooled to 0 °C in an ice bath, before pyridine (21 mL) was added by syringe. After 5 min, the flask was returned to RT. After 1.5 h, the reaction was quenched by adding MeOH (10 mL). The volatiles were removed by rotary evaporation, and the residue was re-dissolved in DMSO (20 mL) and purified by basic RP-HPLC (50-90% MeCN) to afford **1a** (0.850 g, 59%) as a tan solid.

^{1}H NMR (500 MHz, DMSO-d_6) δ 10.86 (s, 1H), 8.17 (s, 1H), 7.84 (s, 1H), 7.70 (d, *J* = 8.9 Hz, 1H), 7.64 (s, 1H), 7.52 (s, 1H), 7.33 – 7.43 (m, 4H), 7.22 – 7.29 (m, 6H), 7.13 – 7.21 (m, 1H), 6.76 – 6.86 (m, 4H), 6.46 (t, *J* = 6.4 Hz, 1H), 5.38 (d, *J* = 4.6 Hz, 1H), 4.58 (dd, *J* = 10.2, 4.5 Hz, 1H), 3.99 (q, *J* = 5.1, 4.6 Hz, 1H), 3.65 (s, 3H), 3.63 (s, 3H), 3.13 – 3.24 (m, 2H), 2.87 (dt, *J* = 12.9, 6.3 Hz, 1H), 2.38 (ddd, *J* = 13.3, 6.7, 4.5 Hz, 1H). ^{13}C NMR (126 MHz, DMSO-d_6) δ 157.98, 157.94, 155.3, 153.5, 147.2, 144.8, 137.5, 135.64, 135.57, 132.6, 129.7, 129.6, 129.5, 127.8, 127.1, 126.6, 126.5, 126.3, 124.7, 122.2, 120.33, 113.2, 113.09, 113.08, 112.7, 110.5, 106.5, 85.51, 85.49, 83.8, 70.7, 64.0, 54.90, 54.86, 39.5 (overlaps with DMSO). HRMS (ESI-TOF) *m/z* calcd for $C_{42}H_{37}N_4O_5^+$ [M + H]$^+$: 677.2764, found: 677.2770.

2-(2′-Deoxy-3′-*O*-[{2-cyanoethyl-*N*,*N*-diisopropyl}phosphoramidyl]-5′-*O*-[4,4'-dimethoxytrityl]-β-D-ribofuranosyl)-2,6-dihydro-2,3,5,6-tetraazacyclopenta[*de*]tetracene (2)

To an oven-dried 50 mL RB flask with a magnetic stir bar was added **1a** (0.83 g, 1.2 mmol), and the vial was sealed and evacuated with argon three times. DCM (23 mL) followed by *N*-methylmorpholine (NMM; 0.54 mL, 4.9 mmol) was added. The reaction mixture was stirred under argon at RT for 30 min. 4-(chloro(diisopropylamino)phosphanyl)butanenitrile (CEP-Cl; 0.54 ml, 2.5 mmol) was added and the reaction

was stirred under argon at RT for 2 hours. The material was evaporated and purified by flash chromatography (SNAP 25 g; EtOAc in heptane: 20-50%, with 1% Et_3N in both eluents) to yield **2** (0.95 g, 88%) as a white solid.

^{1}H NMR (500 MHz, $CDCl_3$) δ 9.45 (s, 1H), 8.37 (s, 1H), 7.64 (dd, *J* = 17.4, 8.3 Hz, 2H), 7.60 (s, 1H), 7.43 – 7.55 (m, 4H), 7.26 – 7.43 (m, 9H), 7.18 – 7.25 (m, 1H), 6.78 – 6.86 (m, 4H), 6.66 (q, *J* = 6.5, 6.0 Hz, 1H), 6.32 (s, 1H), 4.82 – 4.9 (m, 1H), 4.29 (dd, *J* = 9.1, 5.4 Hz, 1H), 4.08 – 4.26 (m, 5H), 3.71 (s, 3H), 3.70 (s, 3H), 3.45 – 3.6 (m, 6H), 3.35 – 3.46 (m, 1H), 2.87 – 2.96 (m, 1H), 2.74 – 2.77 (m, 5H), 2.59 – 2.66 (m, 2H), 2.48 (t, *J* = 6.5 Hz, 1H), 1.50 (d, *J* = 6.5 Hz, 1H), 1.1 – 1.25 (m, 12H).

The purity was confirmed by ^{1}H-NMR. Note that two diastereomers are present due to the chirality of phosphor.

1.3. Spectral data

3-[7-({[*tert*-Butyl(dimethyl)silyl]oxy}methyl)-4-chloro-7*H*-pyrrolo[2,3-*d*]pyrimidin-5-yl]naphthalen-2-amine (3a)

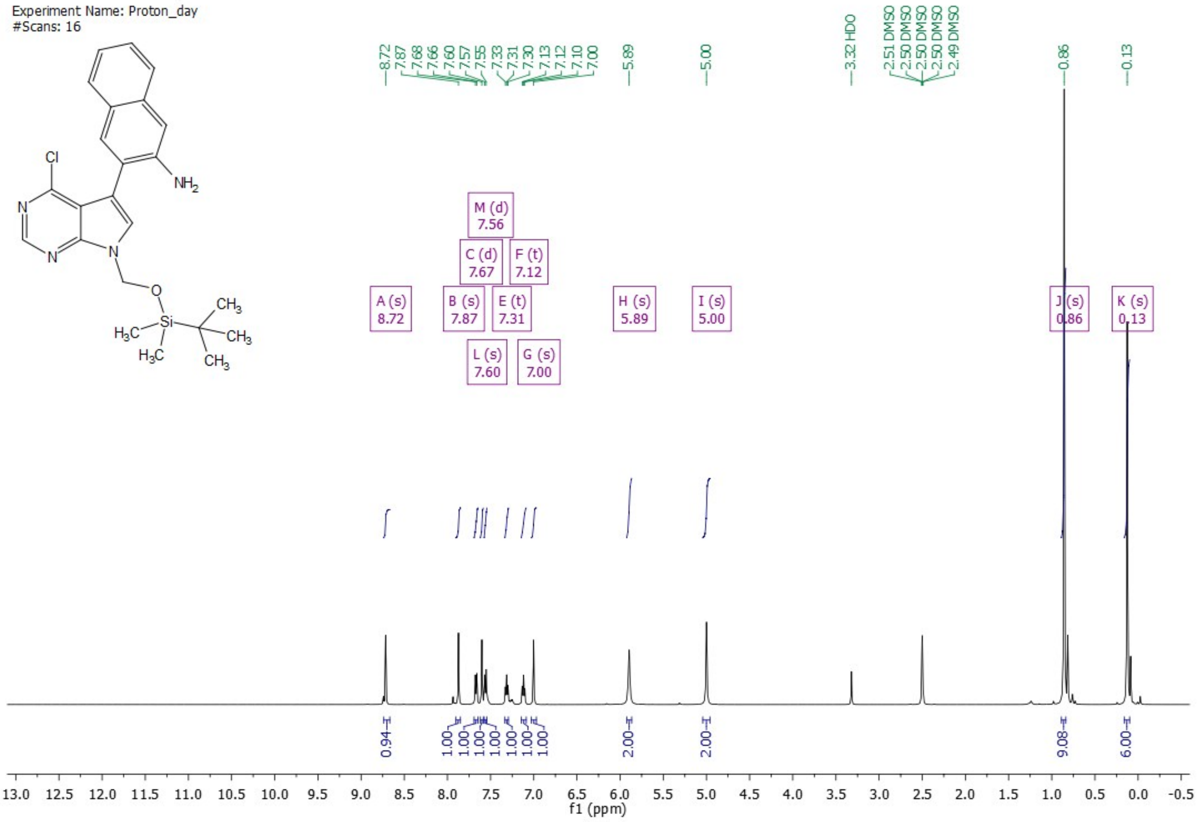

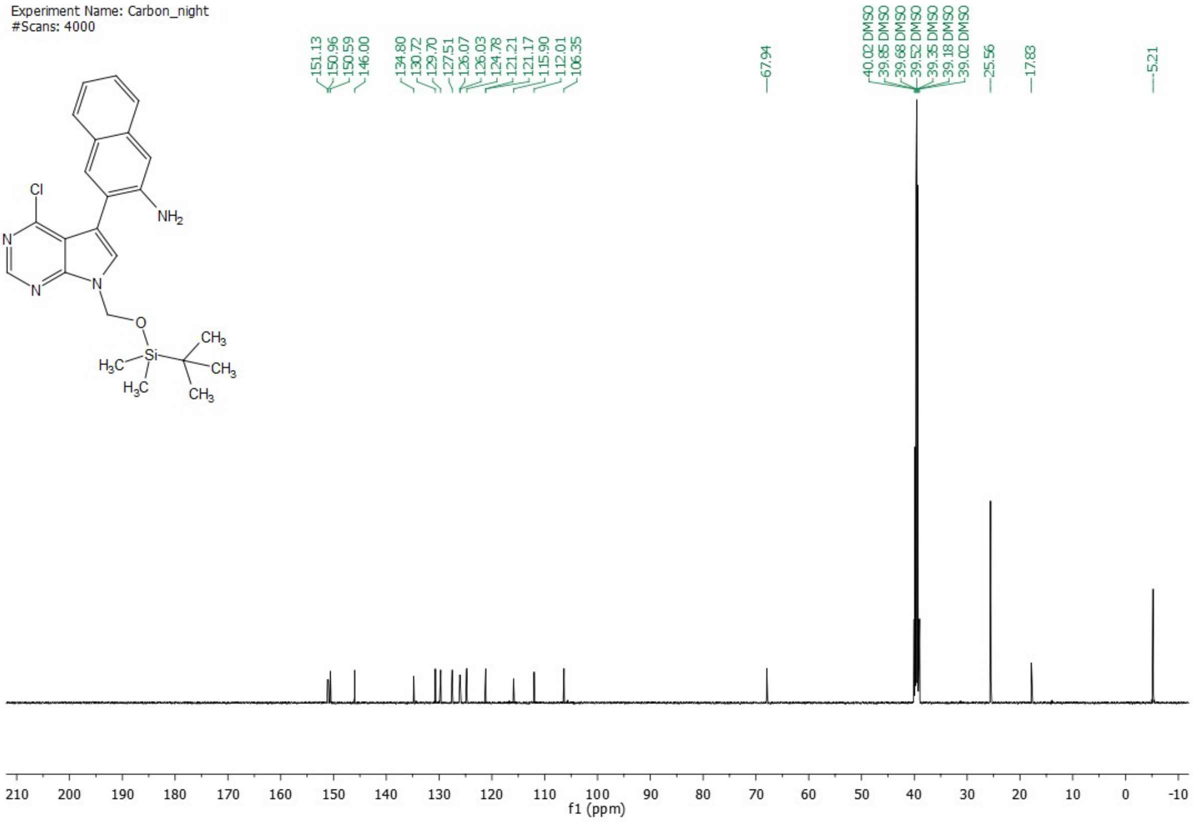

2-({[*tert*-Butyl(dimethyl)silyl]oxy}methyl)-2,6-dihydro-2,3,5,6-tetraazacyclopenta[*de*]tetracene (4)

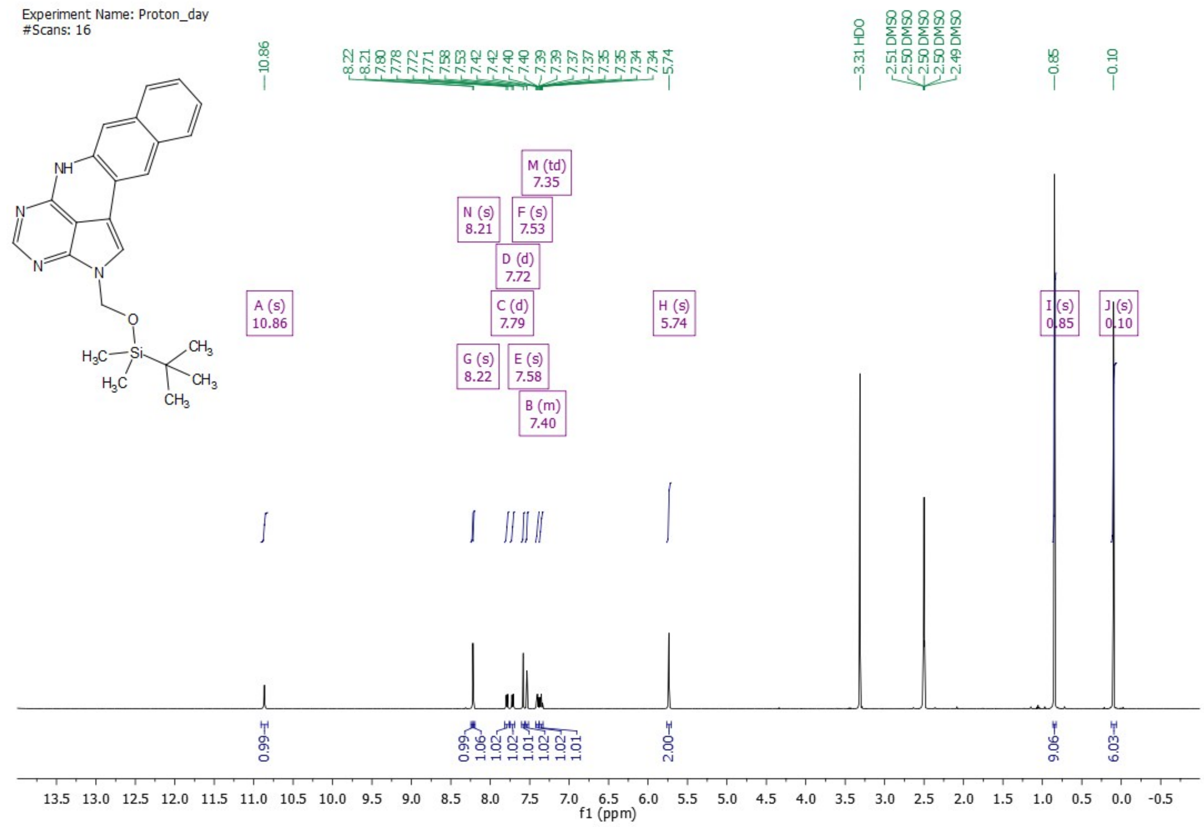

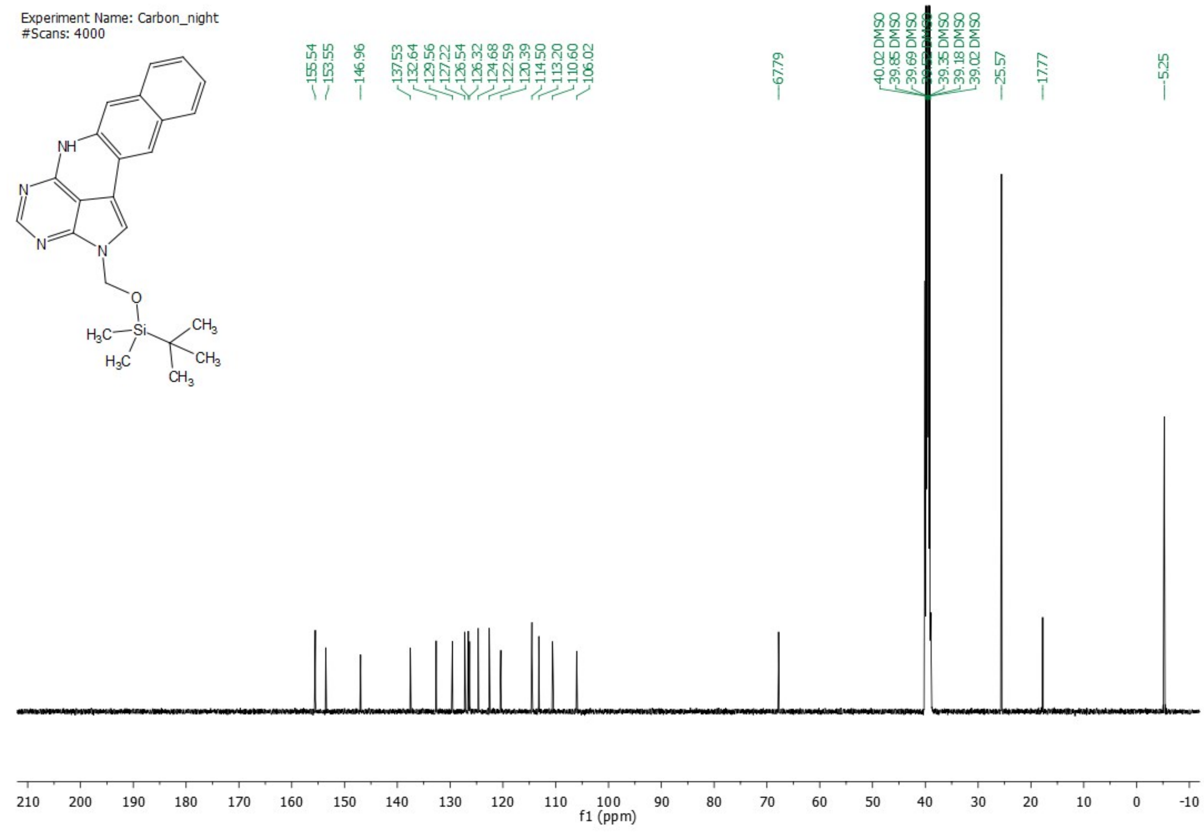

***tert*-Butyl 2-({[*tert*-butyl(dimethyl)silyl]oxy}methyl)-2,3,5,6-tetraazacyclopenta[*de*]tetracene-6(2*H*)-carboxylate (4a)**

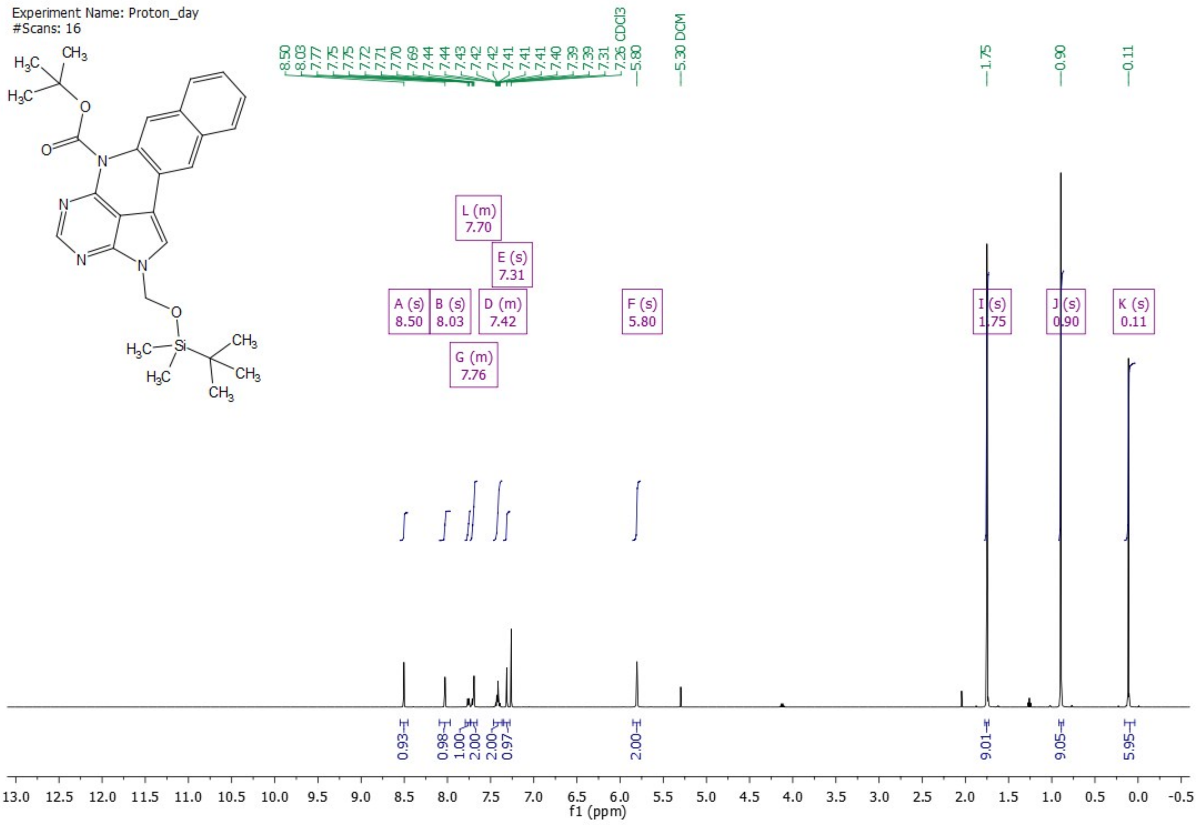

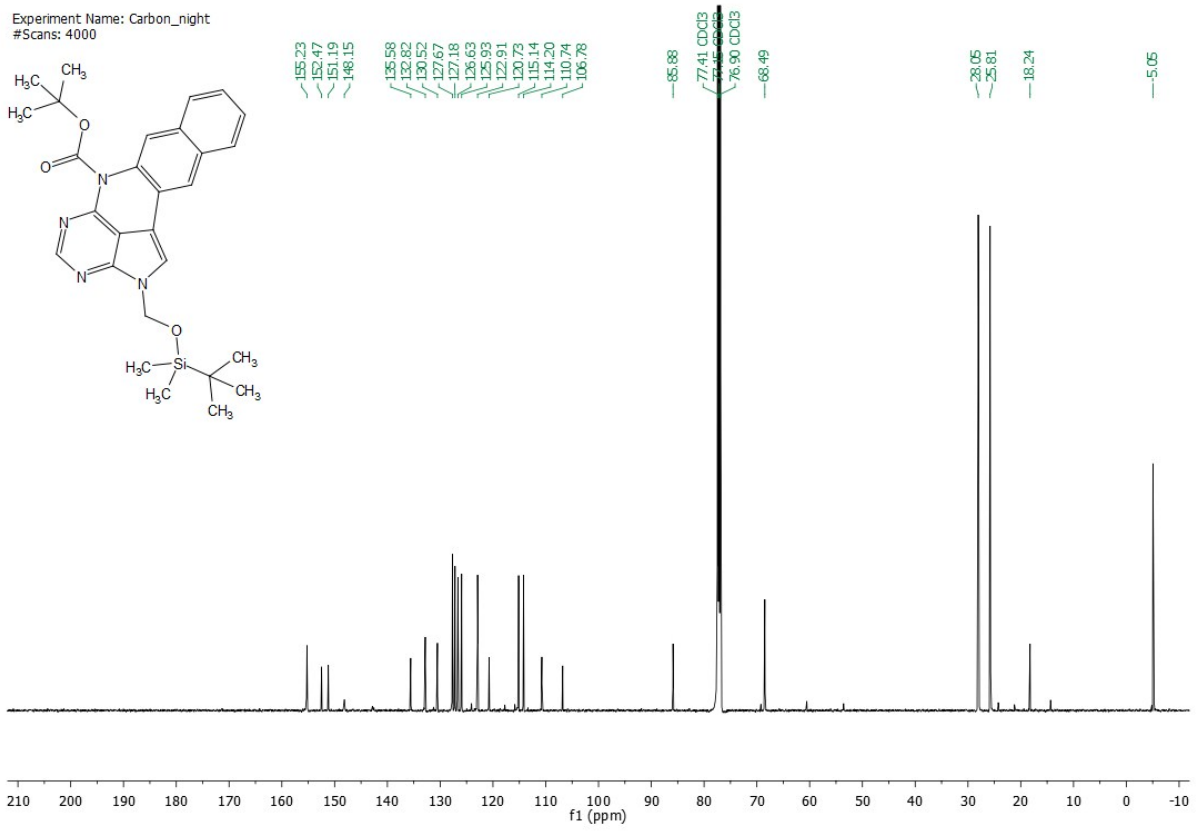

***tert*-Butyl 2,3,5,6-tetraazacyclopenta[*de*]tetracene-6(2*H*)-carboxylate (5)**

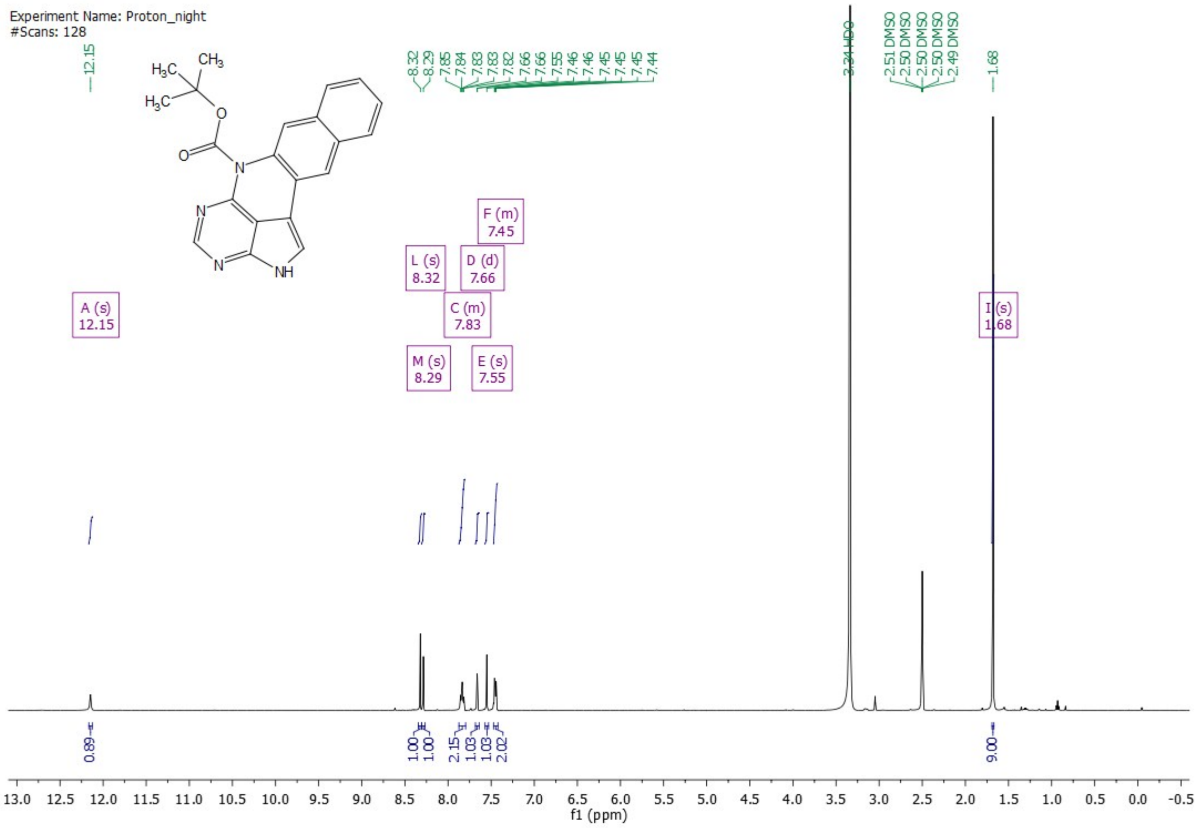

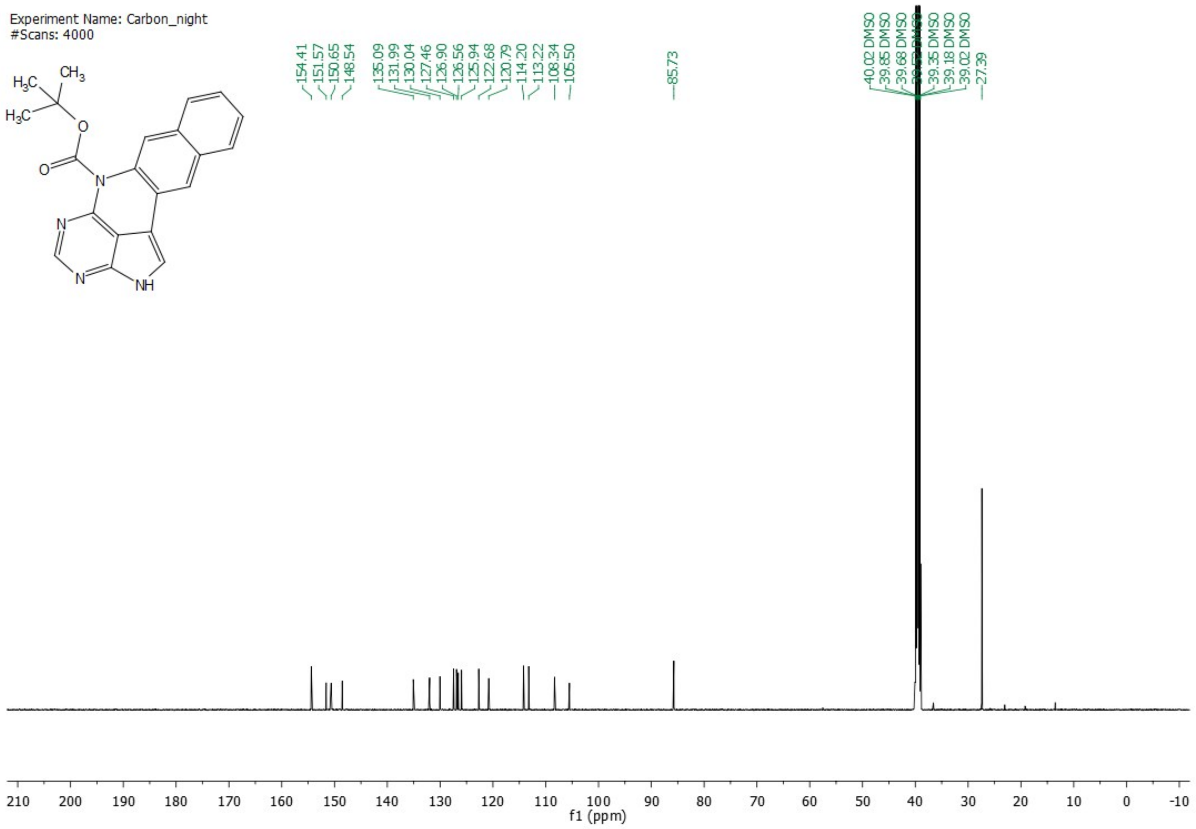

***tert*-Butyl 2-(2′-deoxy-3′-*O*-[4-methylbenzoyl]-5′-*O*-[4-methylbenzoyl]-β-D-ribofuranosyl)-2,3,5,6-tetraazacyclopenta[*de*]tetracene-6(*2H*)-carboxylate (5a)**

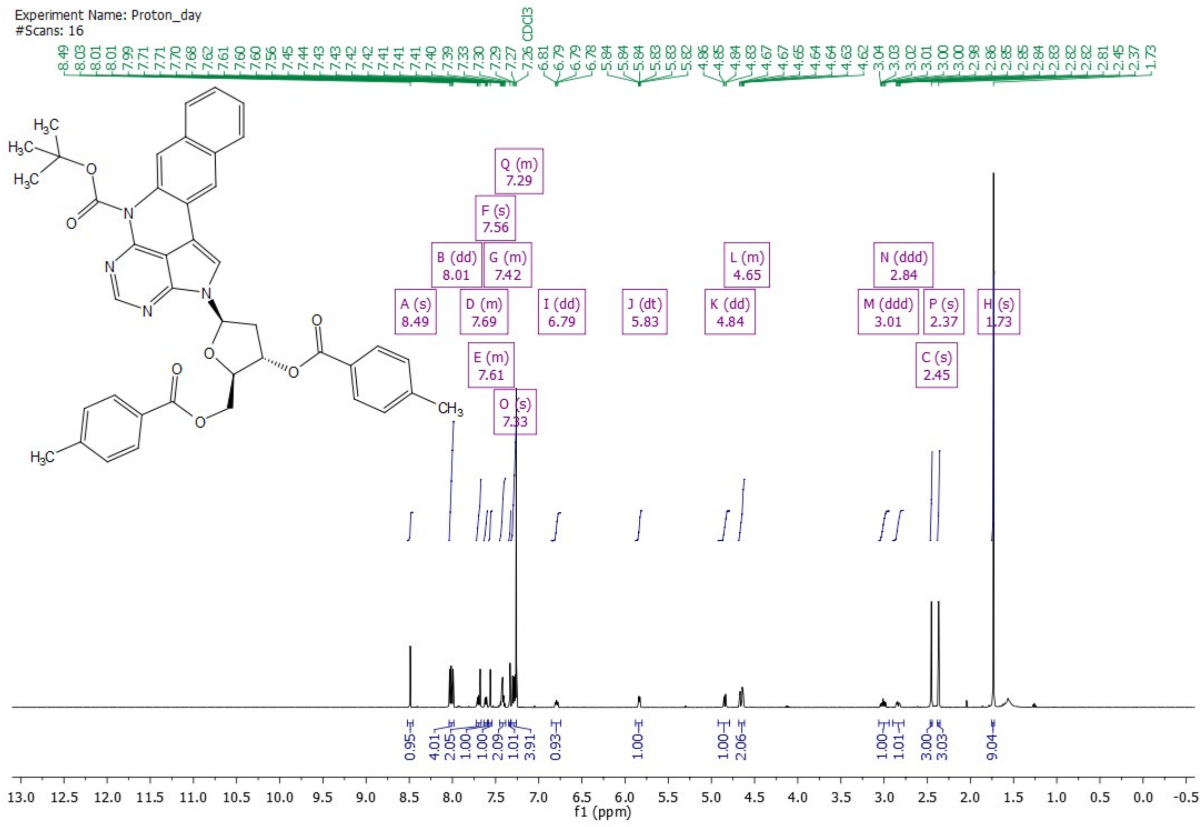

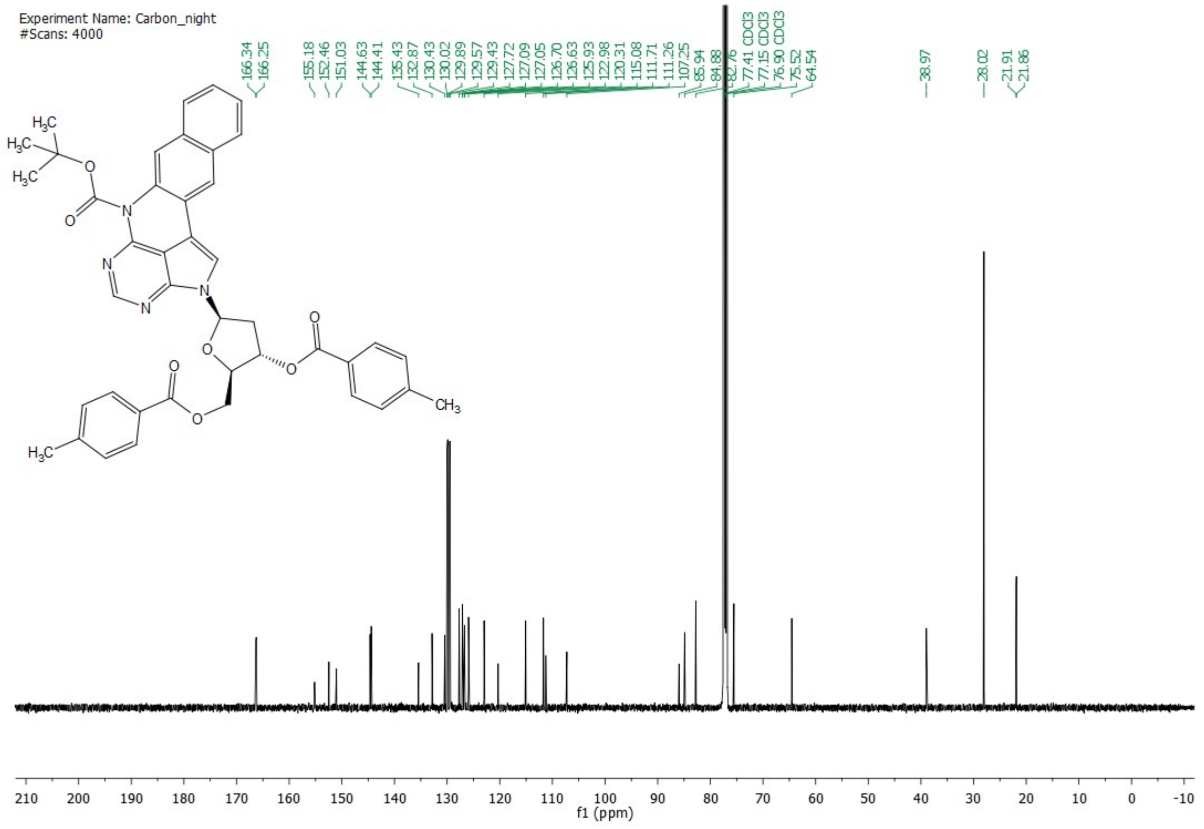

2-(2′-Deoxy-β-D-ribofuranosyl)-2,6-dihydro-2,3,5,6-tetraazacyclopenta[*de*]tetracene (1)

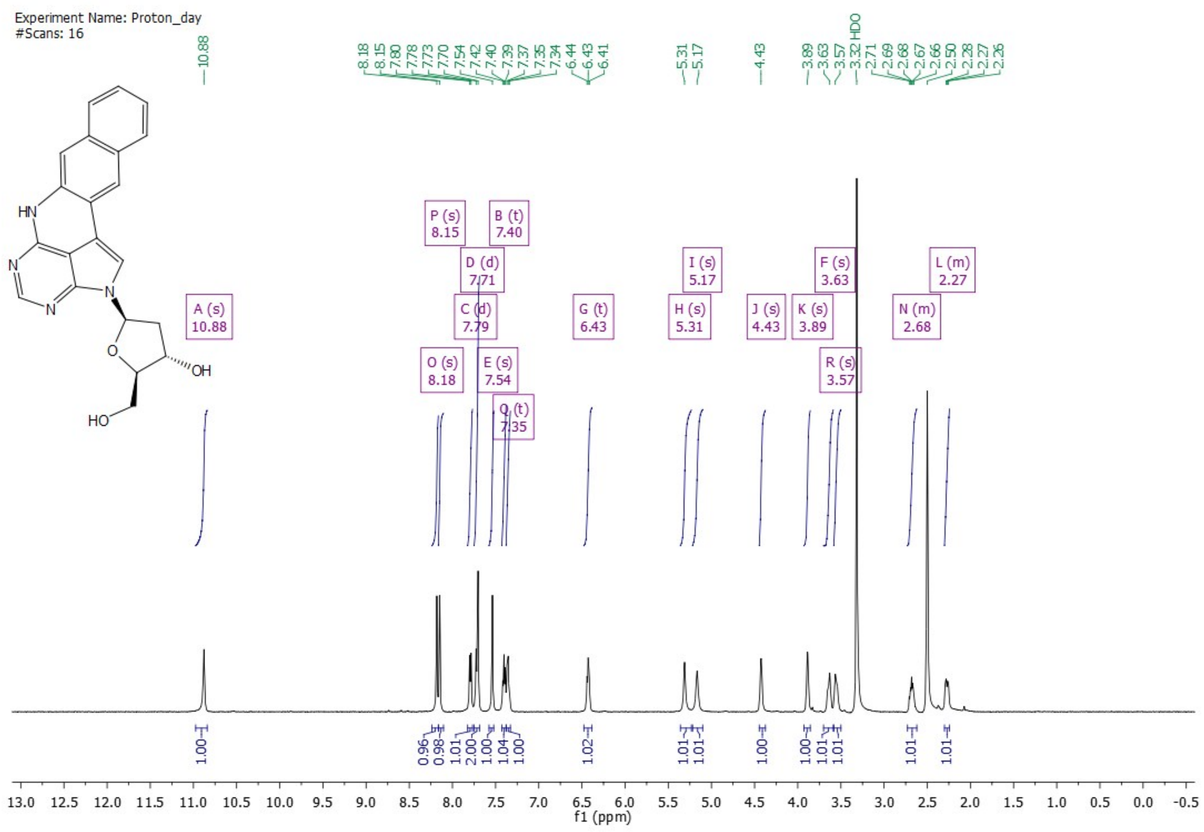

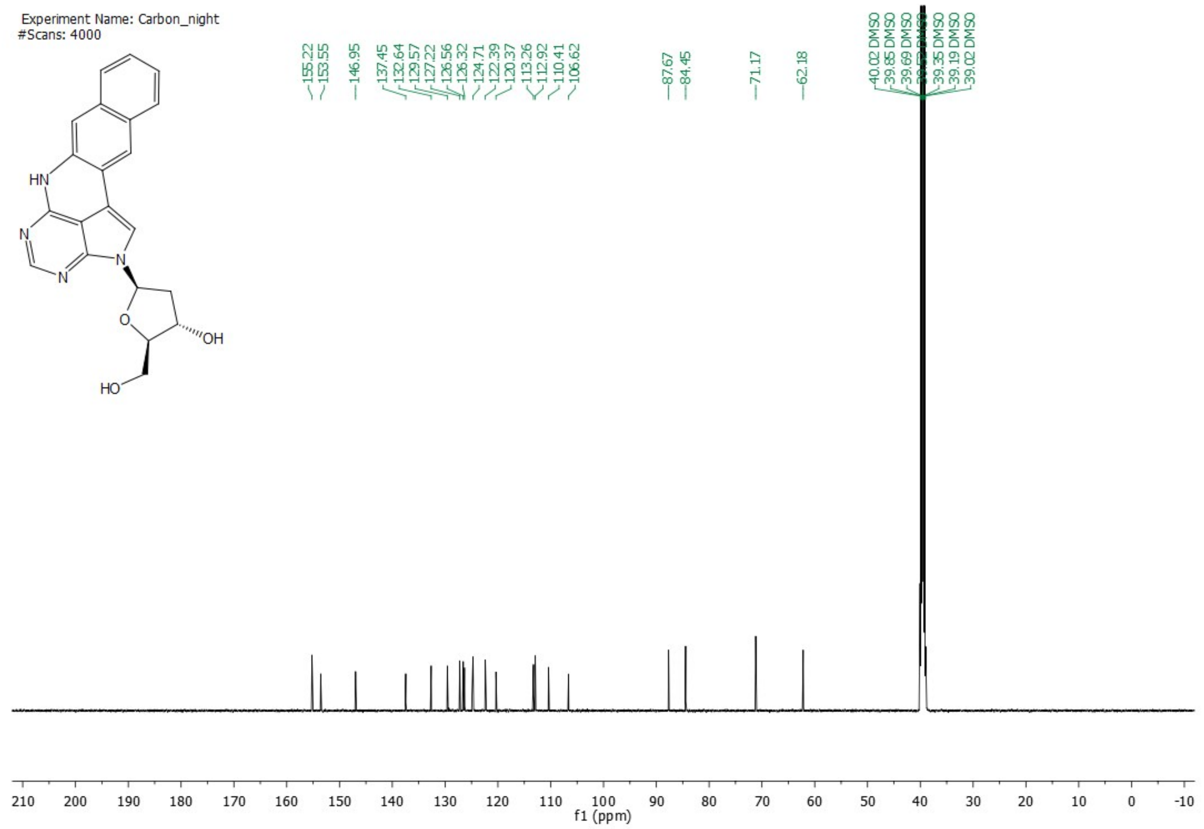

2-{2′-Deoxy-5′-*O*-[4,4'-dimethoxytrityl]-β-D-ribofuranosyl]-2,6-dihydro-2,3,5,6-tetraazacyclopenta[*de*]tetracene (1a)

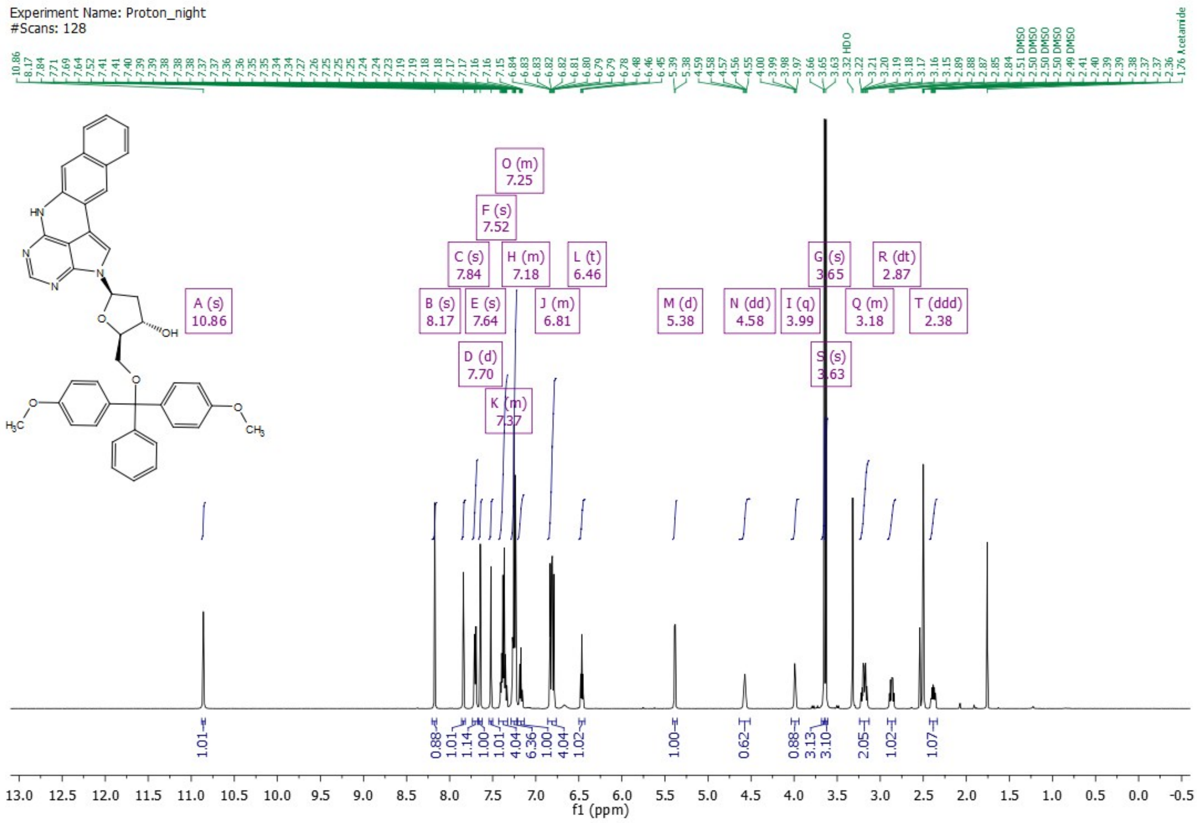

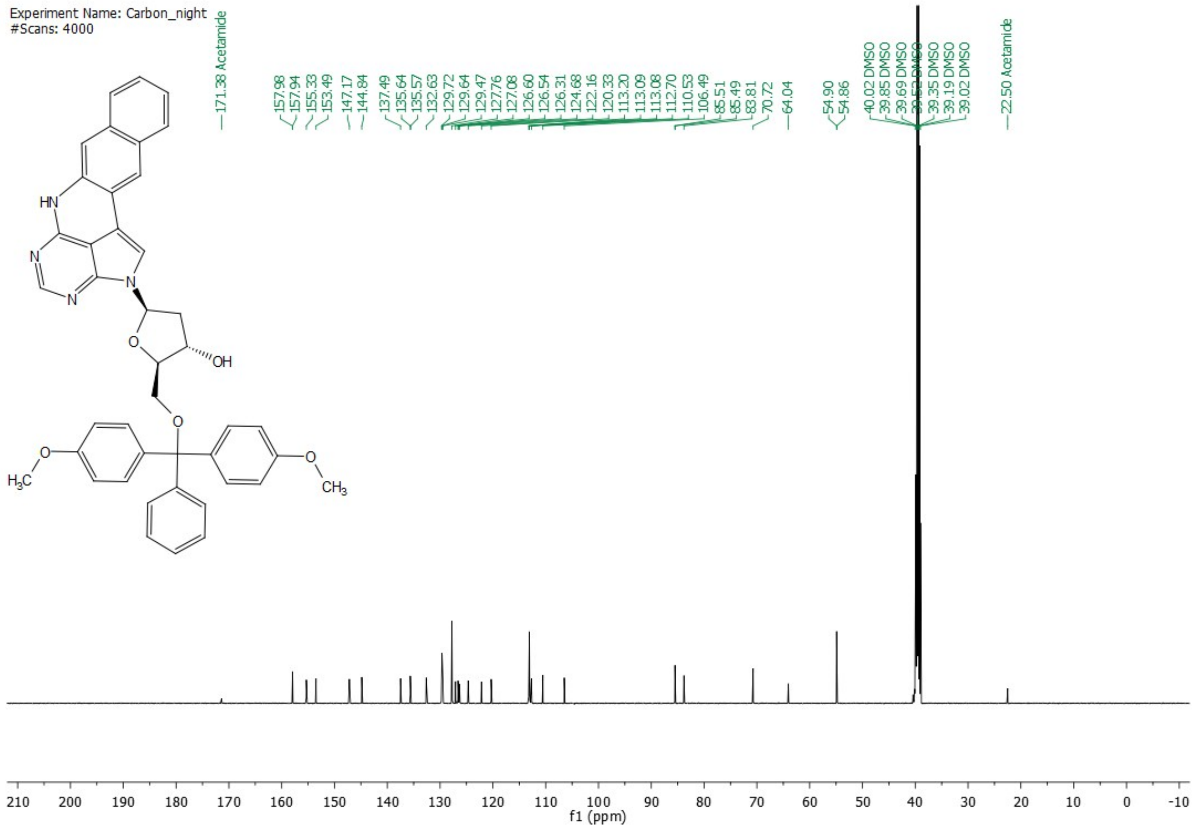

2-(2′-Deoxy-3′-*O*-[{2-cyanoethyl-*N*,*N*-diisopropyl}phosphoramidyl]-5′-*O*-[4,4'-dimethoxytrityl]-β-D-ribofuranosyl)-2,6-dihydro-2,3,5,6-tetraazacyclopenta[*de*]tetracene (2)

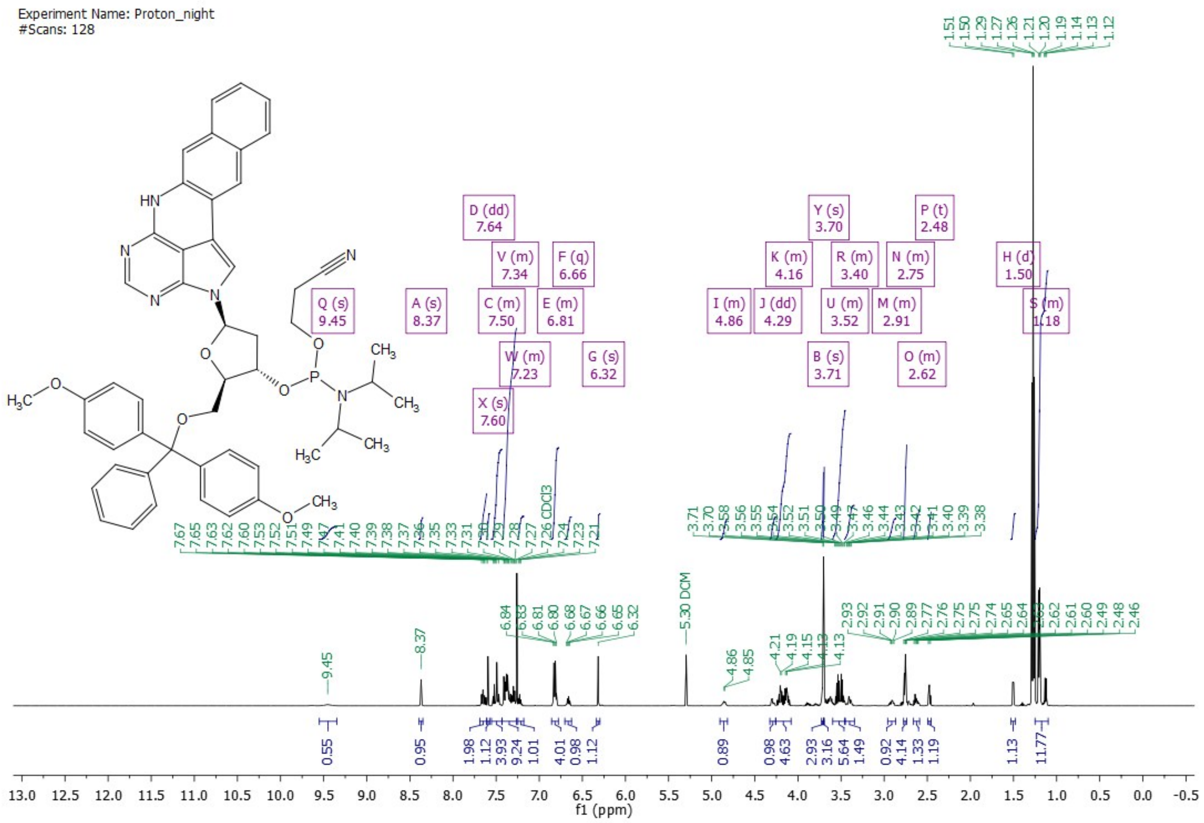

2. Oligonucleotide Synthesis and Analysis

1.1. Oligonucleotide Synthesis

Oligonucleotide synthesis was performed on an Applied Biosystems 394 automated DNA/RNA synthesizer using standard 1.0 µmol phosphoramidite cycle of acid-catalyzed detritylation, coupling, capping and iodine oxidation. All native β–cyanoethyl phosphoramidite monomers were dissolved in anhydrous MeCN to a concentration of 0.1 M immediately prior to use under argon and coupled for 60 s. The pA phosphoramidite monomer **5**, d(pA), was coupled for 10 min. Stepwise coupling efficiencies overall yields were determined by automated trityl cation conductivity monitoring and exceeded 98% for all oligonucleotides synthesized, including oligonucleotides containing d(pA).

Cleavage of oligonucleotides from the solid support and deprotection were achieved by treating with concentrated aqueous ammonia for 1 h at RT followed by heating in a sealed tube for 4 h at 55 °C. Purification of oligonucleotides was carried out by reversed-phase HPLC on a Gilson system using a Brownlee Aquapore column (C_8, 8 mm × 250 mm, 300Å pore diameter) with a gradient of MeCN in an aqueous solution of triethylammonium bicarbonate (TEAB, 0.1M) increasing from 0% to 50% buffer B over 30 min with a flow rate of 4 mL/min (buffer A: 0.1 M TEAB, pH 7.0, buffer B: 0.1 M TEAB, pH 7.0 with 50% MeCN). Elution of oligonucleotides was monitored by UV absorption at 295 or 300 nm. After HPLC purification, oligonucleotides were freeze-dried then dissolved in water without the need for desalting. All oligonucleotides were characterized by electrospray mass spectrometry using a Bruker micrOTOF II focus ESI-TOF MS instrument in ESI-mode. Data were processed using MaxEnt.

2.2. Oligonucleotide Analytical Data

The detailed analytical data for four representative modified oligonucleotides are shown.
AC: 5′-d(CGCAA(pA)CTCG)-3′; *m/z* calcd for $[M{-}H]^-$: 3118.590

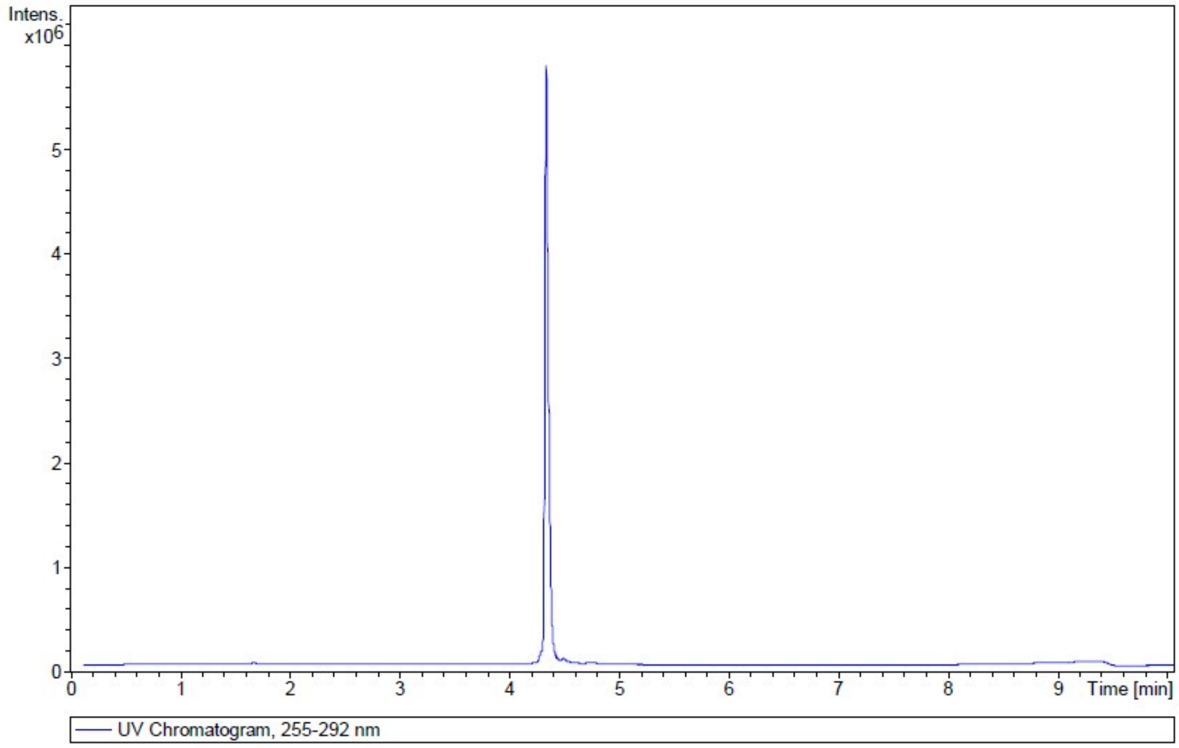

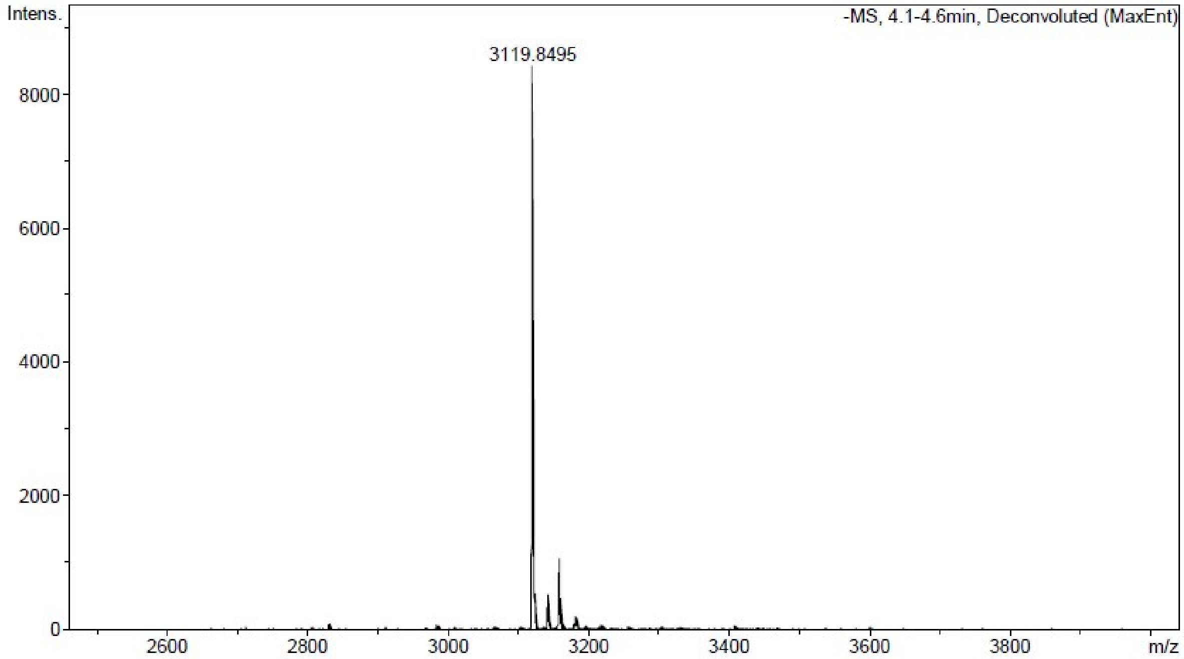

AG: 5′-d(CGCAA(pA)GTCG)-3′; *m/z* calcd for $[M{-}H]^{-}$: 3158.596

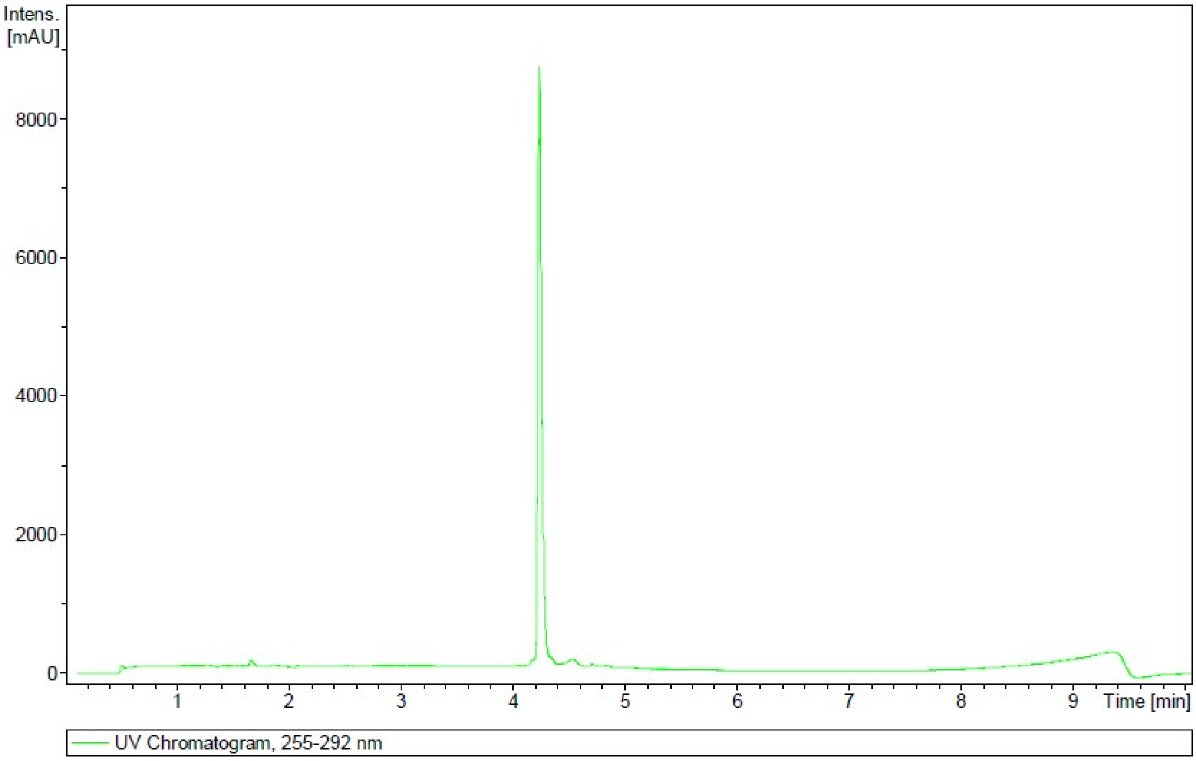

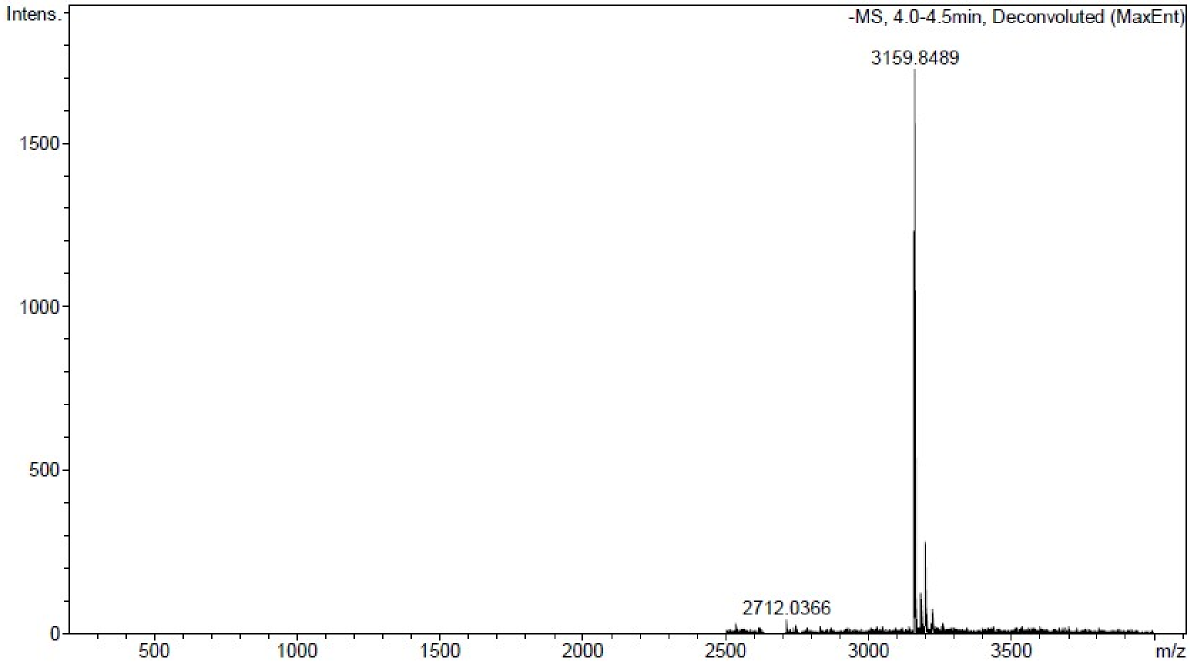

CG: 5′-d(CGCAC(pA)GTCG)-3′; *m/z* calcd for [M–H]⁻: 3133.589

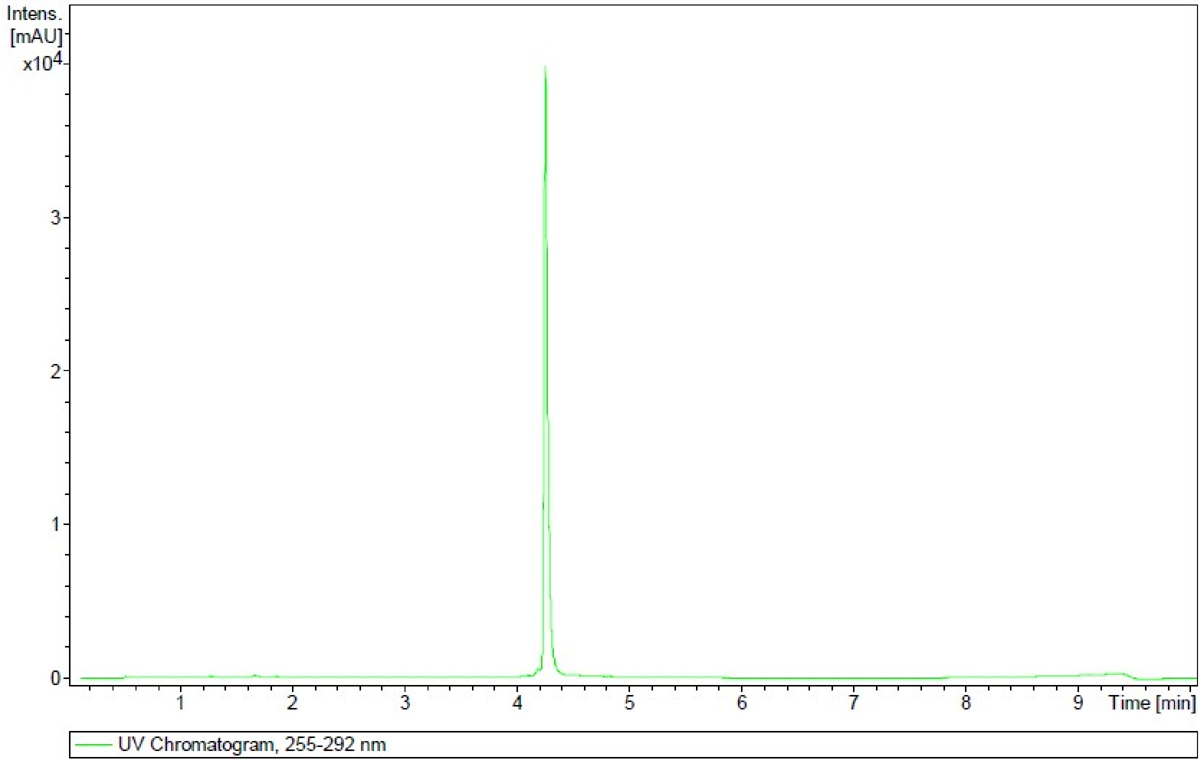

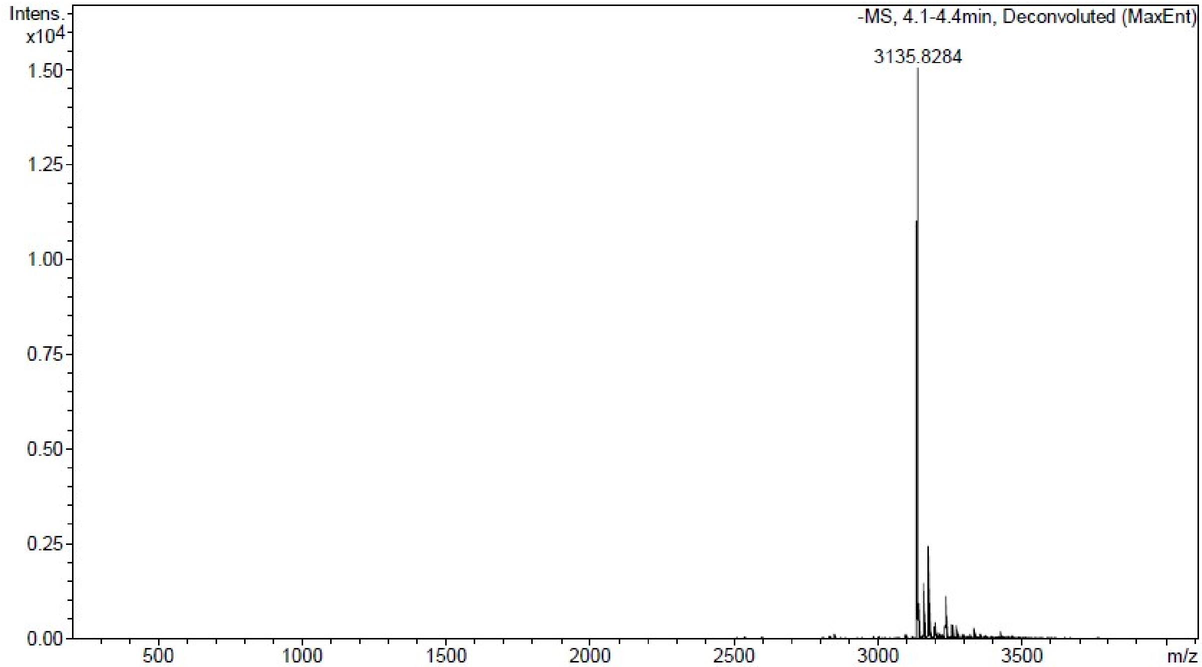

D11: 5′-d(CGA TCA AAA A(pA)A ATT ACG ATT ATA AGG AGG AGG)-3′; *m/z* calcd for $[M–H]^–$: 10393.837

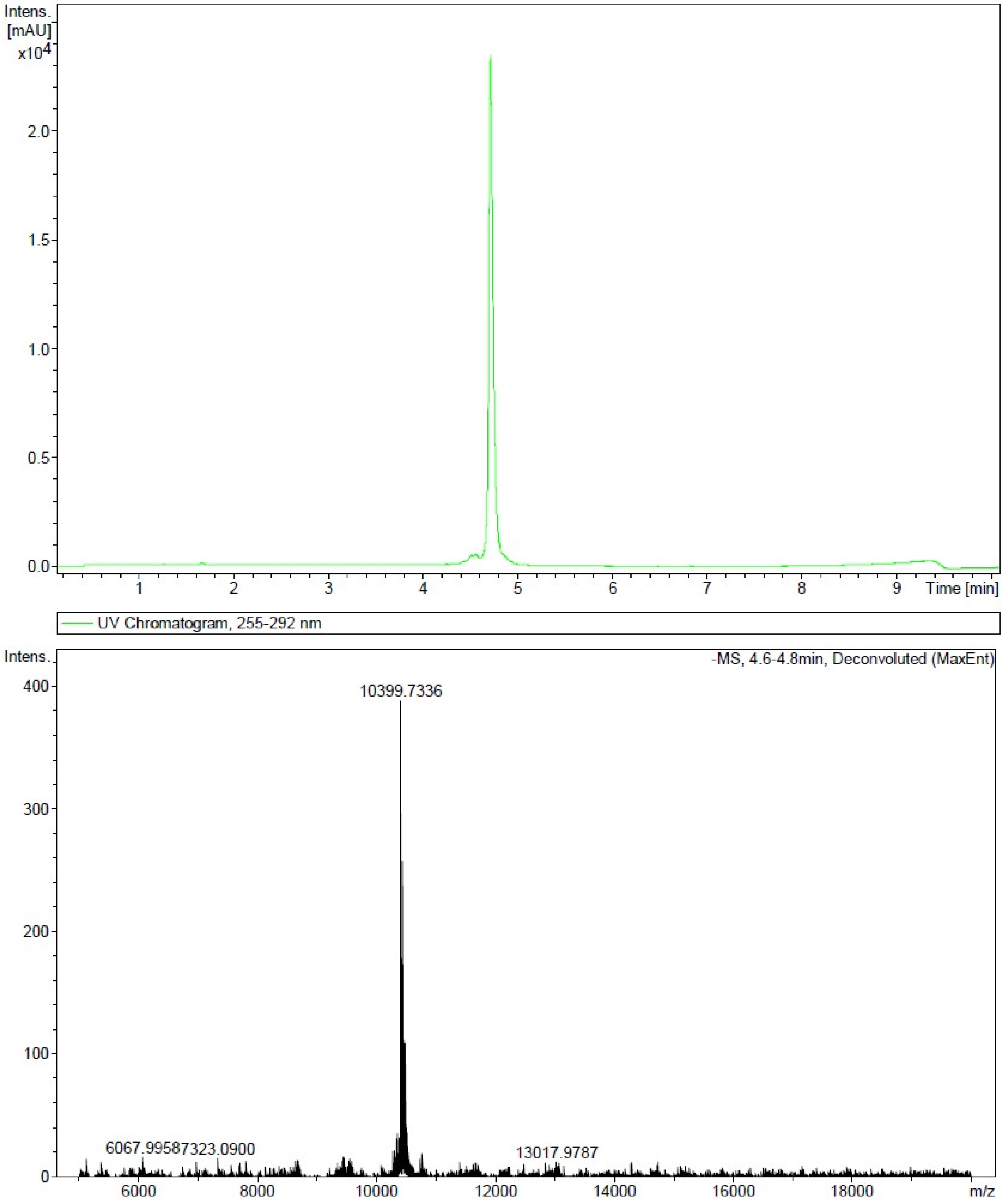

3. Photophysical Experimental Section

3.1. Molar absorptivity and quantum yield of the pA monomer

To determine the molar absorptivity of the pA nucleoside in water, three samples with known concentration of pA (0.5, 0.75 and 1 μM) were prepared from a 1.0 mM stock in DMSO (the final samples contained 2% DMSO in water). Absorption was measured between 200 and 550 nm using a Cary 5000 (Varian Technologies) with a scan rate of 200 nm·min^{-1}. Using the Beer-Lambert law, the molar absorptivity at the maximum of the two lowest energy transitions and at 260 nm was determined. The molar absorptivity of the pA nucleoside monomer in other solvents (DMSO, EtOH, DCM, toluene, MeCN), were measured as above at 2 μM concentration.

3.2. Hybridization of DNA-strands

Sodium phosphate buffer (12.5 mM phosphate, 100 mM NaCl, 123 mM total Na^{+}, pH 7.5) was used for all measurements unless otherwise stated. Before hybridization, absorption spectra between 200 and 550 nm were recorded on a Cary 4000 or Cary 5000 (Varian Technologies) for each of the single strands. The absorption at 260 nm was used for calculating the strand concentration, where the oligonucleotide molar absorptivity at 260 nm was taken as the linear combination of the molar absorptivity of the individual bases at this wavelength, multiplied by 0.9 to account for the effect of base stacking. The values used for the molar absorptivity of each base at 260 nm are: ε(T) = 9300 M^{-1}cm^{-1}, ε(C) = 7400 M^{-1}cm^{-1}, ε(G) = 11800 M^{-1}cm^{-1}, ε(A) = 15300 M^{-1}cm^{-1}, ε(pA) = 22300 M^{-1}cm^{-1} and ε(qA$_{nitro}$) = 12900 M^{-1}cm^{-1}. Hybridization was achieved by mixing each pA modified strand with 15% excess of its complementary strand (to assure full hybridization of the modified strands) at RT followed by heating to 95 °C and, after 10 minutes, cooling to 5 °C over a period of 12 hours.

By measuring absorption on the single-stranded DNA and hybridized duplexes (assuming the concentration is given by the absorption at 260 nm using the molar absorptivities of the DNA bases as stated above) the molar absorptivities of pA and qA$_{nitro}$ in ssDNA and dsDNA at the maximum of the lowest energy transition were determined using the Beer-Lambert law.

3.3. DNA UV-melting and Circular Dichroism (CD)

DNA UV-melting curves were recorded on a Cary 4000 (Varian Technologies) with a programmable multi-cell temperature block, by heating from 10 or 15 °C (depending on T_m) to 85 °C with a rate of 0.5 °C/min and subsequent cooling to 10 or 15 °C at an identical rate. The absorption at 260 nm was recorded every 0.5 °C for two cycles. The duplex concentration was 2 μM in all measurements. The melting temperatures were calculated as the maximum of the first derivative of the UV-melting curves after FFT-filtered smoothing.

Circular dichroism spectra were recorded on a Chirascan CD spectrometer (Applied Photophysics) scanning between 200-450 nm, using an integration time of 0.5 s and four repetitions. The duplex concentration was 4 μM and all spectra were corrected for background contribution.

3.4. Fluorescence measurements

Steady-state emission spectra were recorded on a SPEX Fluorolog 3 (JY Horiba) with an excitation wavelength of 353 nm. The concentration of the single-stranded/duplex samples were 4 μM in all steady-state and lifetime measurements. Emission was recorded between 365 and 690 nm at a scan rate of 600 nm min^{-1}, with the excitation and emission monochromator slit widths set to 1.6 nm. All quantum yields were determined using quinine sulfate (Φ_F = 54.6%) in 0.5 M H_2SO_4 as reference. The quantum yield of the pA nucleoside monomer was measured as above with an excitation wavelength of 368 nm, recording the emission between 378 and 690 nm.

Fluorescence lifetimes were determined using time-correlated single-photon counting (TCSPC). The samples were excited by a PicoQuant pulsed (10 MHz) laser diode emitting at 377 nm and the emission monochromator was set to 408 nm. The counts were collected by a R3809U-50 microchannel-plate photomultiplier tube (Hamamatsu) and fed into a Lifespec multi-channel analyzer (Edinburgh Analytical Instruments) with 2048 channels. 10000 counts were recorded in the top channel. Reconvolutional fitting to mono-, bi- or triexponential functions was performed using Fluofit Pro v.4 software (PicoQuant GmbH). The average lifetimes were amplitude weighted according to equation S1:

$$\langle\tau\rangle = \frac{\sum_i \alpha_i \tau_i}{\sum_i \alpha_i} \tag{S1}$$

where <τ> is the average lifetime, τ_i is the ith lifetime and α_i is the amplitude of the ith lifetime. Measurements were duplicated.
The radiative decay rate (k_r) is calculated using equation S2:

$$k_r = \frac{\Phi_F}{\langle\tau\rangle} \tag{S2}$$

The nonradiative decay rate (k_{nr}) is calculated using equation S3:

$$k_{nr} = \frac{1}{\langle\tau\rangle} - k_r \tag{S3}$$

3.5. FRET measurements

Using the eight sequences shown in Table S5, fifteen duplexes were hybridized: Three with only the donor pA present (at three different positions) and twelve with the donor pA in one strand and the acceptor qA_{nitro} in the opposite strand, resulting in duplexes with 2-13 bases separating the donor and acceptor.
Hybridization was performed as described above, using 15% excess of the unmodified or acceptor strands compared to those containing the donor pA. Steady state emission spectra were measured for all samples as described above, but with an excitation wavelength of 370 nm (at which the ratio of absorption between pA and qA_{nitro} is greatest). Emission was recorded between 374 and 690 nm at a scan rate of 600 nm·s^{-1}, with the excitation and emission monochromator slit widths set to 1.4 nm. Time-resolved decays were measured for all samples using the same settings as described above. The duplex concentration was 4 µM in all measurements. Quantum yields were measured on the three duplexes with only pA present (i.e. no qA_{nitro}) using an excitation wavelength of 353 nm. Emission was recorded between 360 and 650 nm at a scan rate of 600 nm·min^{-1}, with the excitation and emission monochromator slit widths set to 1.5 and 1.8 nm respectively.

3.6. Data evaluation/curve fitting

The average FRET efficiency (E) of the sample was calculated from the measured data using equation S4.

$$E = 1 - \frac{I_{DA}}{I_D} = 1 - \frac{\langle\tau\rangle_{DA}}{\langle\tau\rangle_D} \tag{S4}$$

Where *I* is the integrated steady-state emission and <τ> is the average lifetime (calculated using eq S1 above). DA refers to sample containing both donor and acceptor, D refers to samples containing only donor.
The FRET efficiency is theoretically given by equation S5.

$$E = \frac{R_0^6}{R_0^6 + R_{DA}^6} \tag{S5}$$

where R_0 is the Förster radius, the distance between donor and acceptor that gives 50% FRET efficiency, and R_{DA} is the actual distance between donor and acceptor. R_0 is calculated using equation S6.

$$R_0 = 0.211(J_{DA}\kappa^2 n^{-4}\Phi_D)^{1/6}\,\text{Å} \tag{S6}$$

where J_{DA} is the overlap integral, κ^2 is an orientation factor that takes the orientation between the transition dipole moments of the donor and acceptor into account, n is the refractive index (set to 1.4)[1] and Φ_D is the quantum yield of the donor. κ^2 was approximated by equation S7, assuming straight, B-form DNA and planar, static probes positioned inside DNA.[2]

$$\kappa_{DA} = \cos\left(n_{DA}\beta + \alpha\right) - 3\left(\frac{a * \sin\left(n_{DA}\beta + \alpha\right)}{R_{DA}}\right)^2 \tag{S7}$$

where n_{DA} is the number of base pairs between the donor and acceptor, β is the helical rise angle (34.3°/base pair), α is the phase angle (i.e. the angle between the donor and acceptor transition dipole moments projected onto the base-pair plane) and a is the distance between the center of the chromophore to the center of the DNA helix (3.9 Å). To calculate the distance between donor and acceptor, we use that the average distance between donor and acceptor in the base-pair plane can be expressed as $\frac{2\sqrt{2}}{\pi}a$, which allows R_{DA} to be calculated using equation S8.

$$R_{DA} = \sqrt{\frac{8}{\pi^2}a^2 + (b(n_{DA}+1))^2} \tag{S8}$$

where b is the helical rise (3.4 Å/base pair).

Combining equations S5-S8, the FRET efficiency can be expressed as a function of n_{DA}

$$E = \frac{1}{1 + \frac{\left(\frac{8}{\pi^2}a^2 + \left(b(n_{DA}+1)\right)^2\right)^3}{(0.211)^6 J_{DA} n^{-4}\Phi_D\left(\cos\left(n_{DA}\beta+\alpha\right) - 3\left(\frac{a\sin\left(n_{DA}\beta+\alpha\right)}{\sqrt{\frac{8}{\pi^2}a^2 + (b(n_{DA}+1))^2}}\right)^2\right)^2}} \tag{S9}$$

An in-house made MATLAB program was used to fit a theoretical FRET-efficiency curve to the measured data (using an average QY of 24.0%), and returning fitted values of J_{DA} and α.

4. TIRF experimental setup

4.1. Materials

1-Palmitoyl-2-oleoyl-*sn*-glycero-3-phosphocholine (POPC) and 1,2-dioleoyl-*sn*-glycero-3-phosphoethanol-amine-*N*-(Cap biotinyl) (sodium salt) (biotin-cap-DOPE) and Lissamine™ Rhodamine B 1,2-dihexadecanoyl-*sn*-glycero-3-phosphoethanolamine, triethylammonium salt (Rhodamine DHPE) was were purchased from Avanti Polar Lipids (USA). Cholesteryl TEG (chol)-modified DNA sequences CholC (5'-chol-CCC GAA CTC GTG GCT-3') and CholD (5'-CGA TTT TGC GCG ATT TTG CGC GAT TTT GCG CGA TTT TGC GCG ATT TTG CGA GCC ACG AGT TCC CC-chol-3') were purchased from Eurogentec (Belgium).

4.2. Vesicle preparation

Lipid vesicles were prepared by the lipid film hydration and extrusion method.[3] Briefly, POPC, biotin-cap-DOPE and Rhodamine-DHPE lipids dissolved in $CHCl_3$ or MeOH were pipetted into a round bottom flask with a desired molar ratio (98:1:1) to control the lipid composition. The lipid mixture was first dried under a gentle N_2

stream to form a lipid film and further dried in vacuum (minimum 2 hrs). This thin dried lipid film was then rehydrated in 1 mL phosphate buffered saline (PBS: 10 mM phosphate buffer, 2.7 mM KCl, 137 mM NaCl, pH 7.4) by vortexing, for a total lipid concentration of 1 mg/mL. The resulting suspension was extruded 11 times through a 30 nm polycarbonate filter at a pressure of 1 bar and stored at 4 °C until usage.

4.3. Size distribution and concentration of vesicles with Nanoparticle Tracking Analysis

The size distribution of the extruded vesicles was determined with a Nanoparticle Tracking Analysis (NTA) LM10 device equipped with a 488 nm laser (Malvern, UK) and analyzed using the NTA software version 3.1. The camera and analysis settings were optimized to enable size distribution and concentration according to the manufacturer recommendations: Camera shutter: 696, camera gain: 73, detection threshold: 2, blur and minimum track length: automatic, minimum expected particle size: 30 nm. The lipid vesicle suspension, measured at a lipid concentration of 0.2 µg/mL, had a vesicle concentration of 3.18×10^{9} $\pm0.04\times10^{9}$ particles/mL and a size distribution with a mean diameter of 91.9 ±0.7 nm and a full width at half maximum of 46 nm.

4.4. Vesicle functionalization with pA-cholesterol-DNA complex

Prior to observation in the microscope, the vesicles were modified with pA-cholesterol-DNA complex. First, the pA-cholesterol-DNA complex was formed by hybridizing the pA-modified sequence AA to a pair of cholesterol-terminated DNA strands (CholC and CholD, the latter containing 5 binding sites for the AA sequence) in a molar ratio of 5:1:1, in a 1:4 volume ratio of Milli-Q water and PBS, for 2 hours. The lipids vesicles were then incubated with the pA-cholesterol-DNA-complex at a 1:1 volume ratio and a molar ratio of 500 pA-cholesterol-DNA complex per vesicle for 30 minutes (final vesicle concentration: 5 nM), leading to an irreversible self-incorporation of the pA-cholesterol-DNA complex into the vesicle lipid membrane.[4]

4.5. Total internal reflection fluorescence (TIRF) microscopy and image analysis

All fluorescence micrographs were acquired with an inverted microscope (Nikon Ti Eclipse, Japan) equipped with TIRF system, a 60× magnification (Numerical aperture (NA) = 1.49) oil immersion objective (Nikon Corporation, Tokyo, Japan), TRITC (Rhodmaine-DHPE) and DAPI (pA probe) filter cubes (Nikon Corporation), perfect focus S6 system and an Andor DU-897 X-3530 EMCCD camera (Andor Technology, Belfast, Northern Ireland). A mercury lamp (Intensilight C-HGFIE; Nikon Corporation) connected to the microscope via an optical fiber served as illumination source. Micrographs containing 512 × 512 pixels (0.41 µm/pixel) were acquired with an exposure time of 500 ms.

In TIRF mode, only vesicles close to the surface (~100 nm) are excited by the evanescent field. This enables detection of individual vesicles bound to the surface even in the presence of a bulk concentration of suspended, unbound vesicles.

4.6. Sample holder and binding of vesicles to surface

Vesicles decorated with pA-cholesterol-DNA complex were unspecifically bound to borosilica coverslips forming the bottom of wells made of polydimethylsiloxane (PDMS, Sylgard 184, Dow Corning, Midland, MI). Briefly, borosilicate coverslips (no. 1; Brand, Germany) were cleaned in boiling 1% Liquinox (Alconox) in water for 30 min, rinsed thoroughly with Milli-Q water then stored in Milli-Q for a maximum of 2 weeks before usage. PDMS wells were formed by first casting PDMS polymer in a 1-3 mm thick sheet which was then cut to fit the geometry of the coverslip and punctured with a hole-punch to create wells. The perforated PDMS sheet

was let to adhere to the cleaned borosilica surface to form wells. The wells were then filled with 20 µL PBS buffer and the coverslip was mounted on the sample holder of the microscope.

Fluorescently labeled vesicles decorated with pA-cholesterol-DNA complex were then injected in the well (10 µL) to reach a final vesicle concentration of 50 fM. The binding of vesicles was monitored in fluorescence mode in the TRITC filter channel, corresponding to the excitation and collection wavelength of the rhodamine fluorophore. The vesicles were unspecifically adsorbed to the glass surface for approximately 30 seconds to reach adequate surface coverage (~100 particles in field of view) followed by thorough rinsing with PBS buffer to stop the binding of unbound vesicles. Fluorescence signal of the pA probe was observed with DAPI filter.

5. Additional figures, tables, charts and notes

5.1. Chart S1

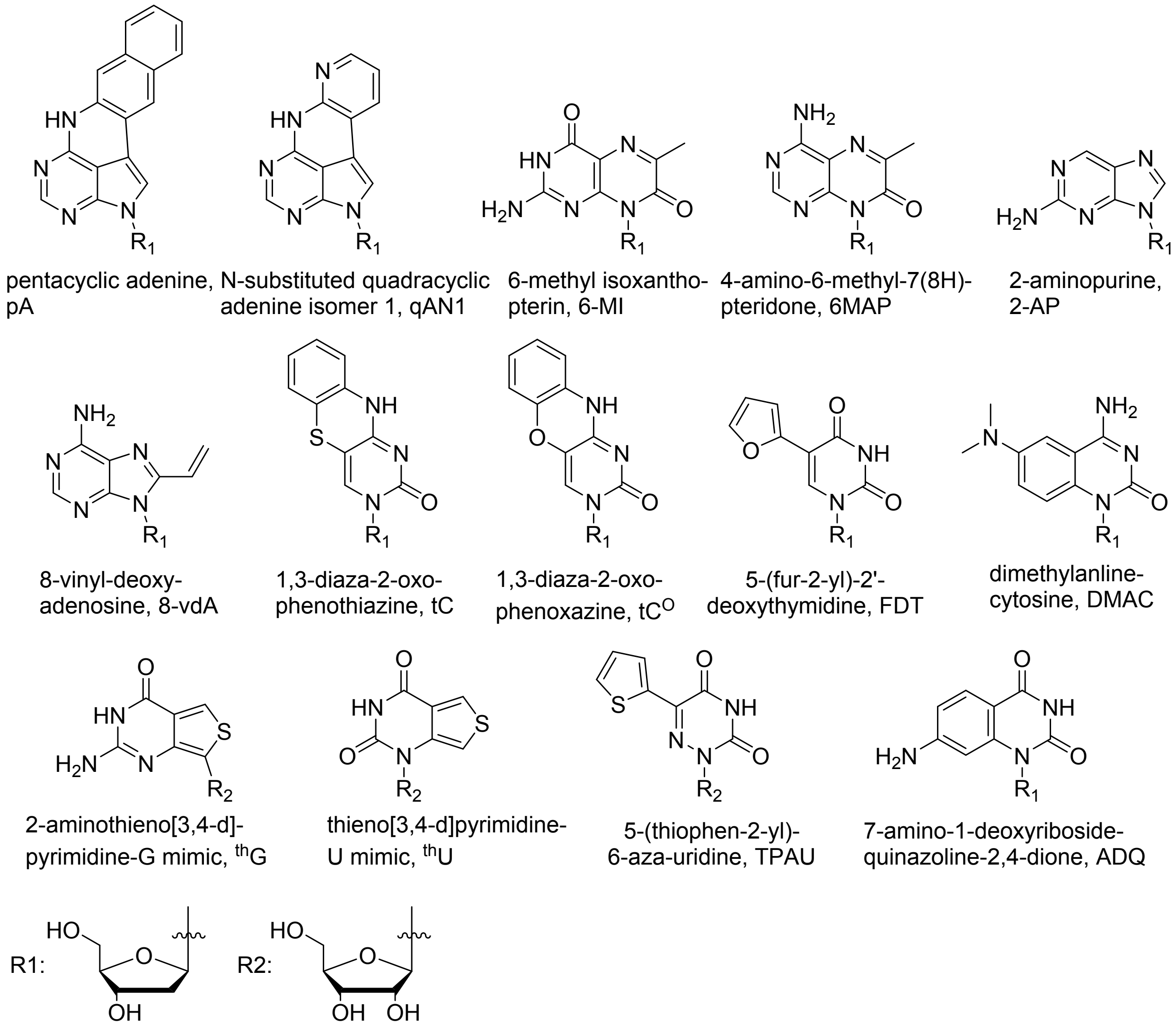

Chart S1. Fluorescent nucleobase analogs listed in Table 5.

5.2. Chart S2

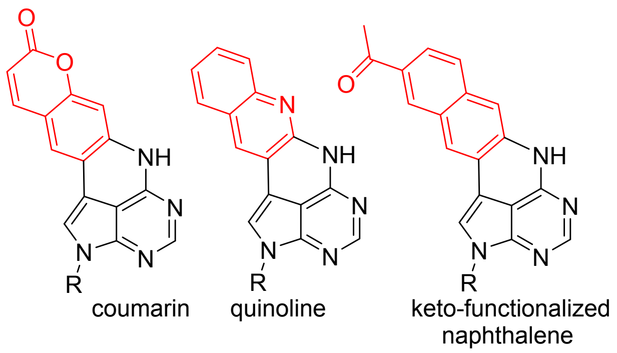

Chart S2. Pentacyclic adenine analogs with a coumarin, a quinoline or a keto-functionalized naphthalene moiety attached. R denotes the sugar–phosphate backbone

5.3. Figure S1

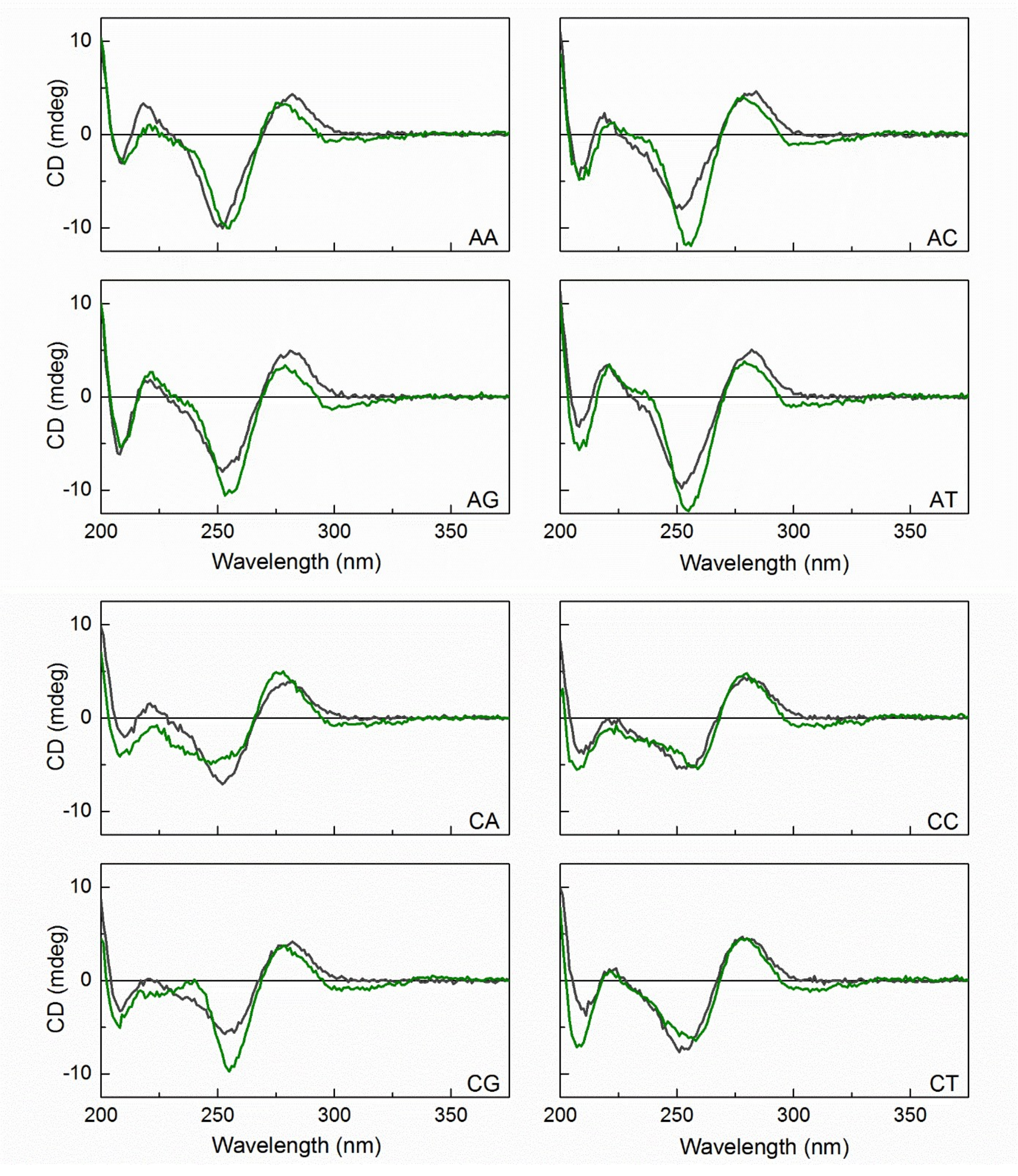

Figure S1. CD spectra of duplexes (AA, AC, AG, AT, CA, CC, CG and CT) with pA (green) and with A (black). Duplexes were formed as described in the experimental section, and were measured at RT in phosphate buffer, pH 7.5, 123 mM Na^+.

5.4. Figure S2

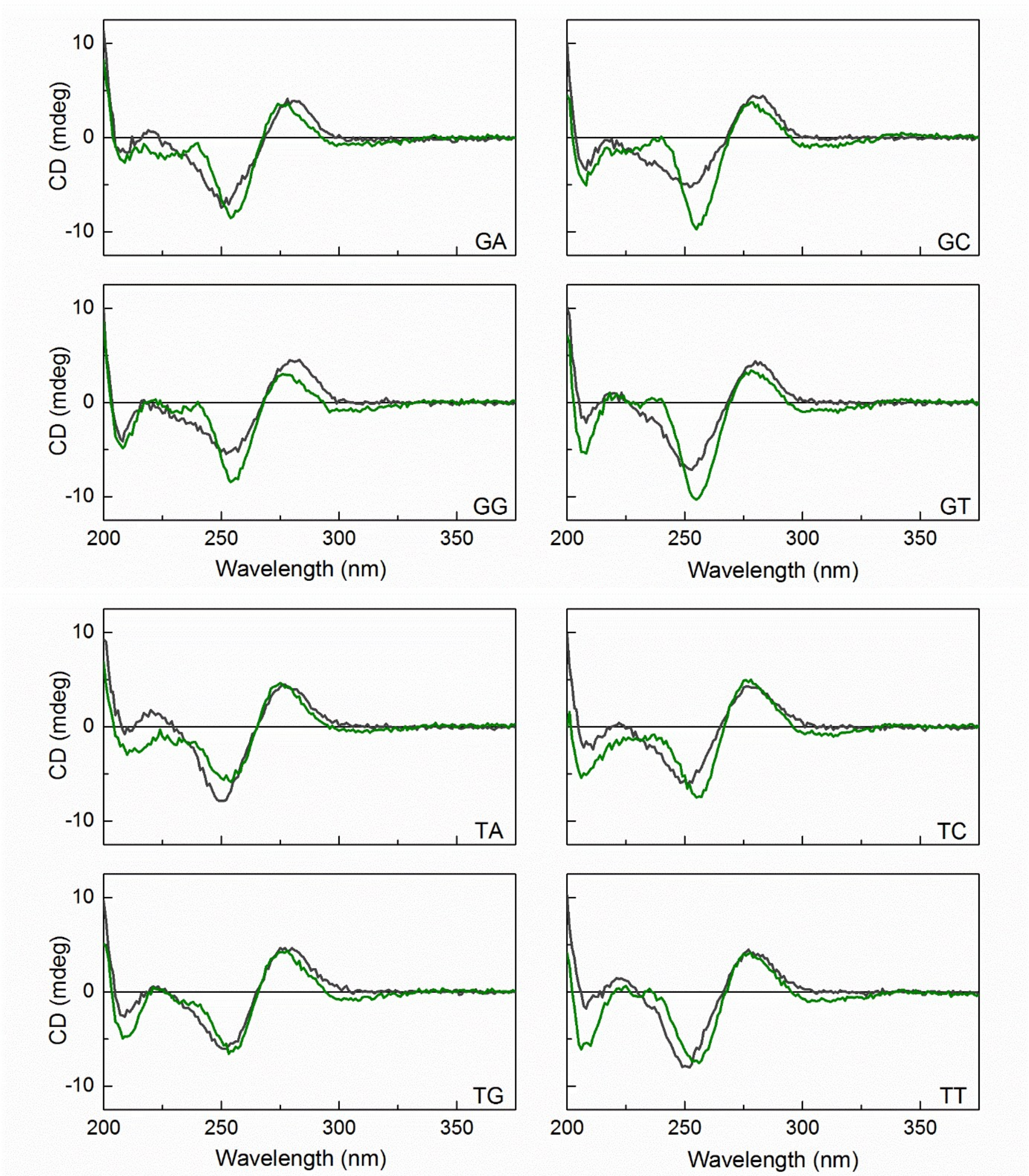

Figure S2. CD spectra of duplexes (GA, GC, GG, GT, TA, TC, TG and TT) with pA (green) and with A (black). Duplexes were formed as described in the experimental section, and were measured at RT in phosphate buffer, pH 7.5, 123 mM Na^+.

5.5. Table S1

Table S1. Melting temperatures of pA-modified duplexes (T_m^{pA}), unmodified duplexes (T_m^{A}) and the difference (ΔT_m) between them.

Sequence name[a]	DNA sequence[b]	T_m^{pA} (˚C)	T_m^{A} (˚C)	ΔT_m (˚C)
AA	5′-d(CGCA**A(pA)A**TCG)-3′	40.8 ±0.1	43.5 ±0.2	-2.7 ±0.2
AC	5′-d(CGCA**A(pA)C**TCG)-3′	45.3 ±0.3	47.1 ±0.2	-1.8 ±0.3
AG	5′-d(CGCA**A(pA)G**TCG)-3′	42.4 ±0.3	45.9 ±0.2	-3.5 ±0.4
AT	5′-d(CGCA**A(pA)T**TCG)-3′	44.8 ±0.1	43.4 ±0.1	1.4 ±0.1
CA	5′-d(CGCA**C(pA)A**TCG)-3′	49.9 ±0.1	46.5 ±0.1	3.4 ±0.1
CC	5′-d(CGCA**C(pA)C**TCG)-3′	54.6 ±0.1	50.3 ±0.1	4.3 ±0.2
CG	5′-d(CGCA**C(pA)G**TCG)-3′	52.2 ±0.2	49.5 ±0.1	2.7 ±0.2
CT	5′-d(CGCA**C(pA)T**TCG)-3′	53.2 ±0.1	47.3 ±0.1	5.9 ±0.1
GA	5′-d(CGCA**G(pA)A**TCG)-3′	43.0 ±0.2	45.3 ±0.2	-2.3 ±0.3
GC	5′-d(CGCA**G(pA)C**TCG)-3′	48.4 ±0.2	49.2 ±0.2	-0.8 ±0.3
GG	5′-d(CGCA**G(pA)G**TCG)-3′	47.6 ±0.1	48.1 ±0.2	-0.5 ±0.2
GT	5′-d(CGCA**G(pA)T**TCG)-3′	46.4 ±0.2	45.4 ±0.2	1.0 ±0.3
TA	5′-d(CGCA**T(pA)A**TCG)-3′	43.0 ±0.1	41.1 ±0.3	1.9 ±0.3
TC	5′-d(CGCA**T(pA)C**TCG)-3′	46.1 ±0.2	43.7 ±0.1	2.4 ±0.2
TG	5′-d(CGCA**T(pA)G**TCG)-3′	45.3 ±0.2	43.6 ±0.1	1.7 ±0.2
TT	5′-d(CGCA**T(pA)T**TCG)-3′	45.8 ±0.3	40.6 ±0.1	5.2 ±0.3

[a] Sequences are named by the bases neighboring pA on the 5′- and 3′-sides, respectively. [b] Unmodified samples contain an adenine instead of pA. Duplexes were formed by hybridization with the complementary strand as described in the experimental section. The melting temperatures were calculated as the maximum of the first derivative of the UV-melting curves and are reported with the standard error of the mean.

5.6. Table S2

Table S2. Melting temperatures (T_m) of matched and mismatched pA duplexes as well as the difference (ΔT_m) between them.

Sample name[a]	T_m (˚C)[b]	ΔT_m (˚C)
CT (T)	53.2 ±0.1	
CT (A)	42.4 ±0.1	-10.8 ±0.1
CT (C)	45.7 ±0.1	-7.5 ±0.1
CT (G)	44.9 ±0.1	-8.3 ±0.1
GA (T)	43.0 ±0.2	
GA (A)	37.4 ±0.1	-5.6 ±0.3
GA (C)	40.4 ±0.1	-2.6 ±0.3
GA (G)	35.8 ±0.3	-7.2 ±0.4
TA (T)	43.0 ±0.1	
TA (A)	35.6 ±0.1	-7.4 ±0.2
TA (C)	37.8 ±0.1	-5.2 ±0.2
TA (G)	36.2 ±0.2	-6.8 ±0.2

[a] The base opposite pA is given in parenthesis. Duplexes were formed by hybridization with the complementary strand as described in the experimental section. [b] The melting temperatures were calculated as the maximum of the first derivative of the UV-melting curves, and are reported with the standard error of the mean.

5.7. Figure S3

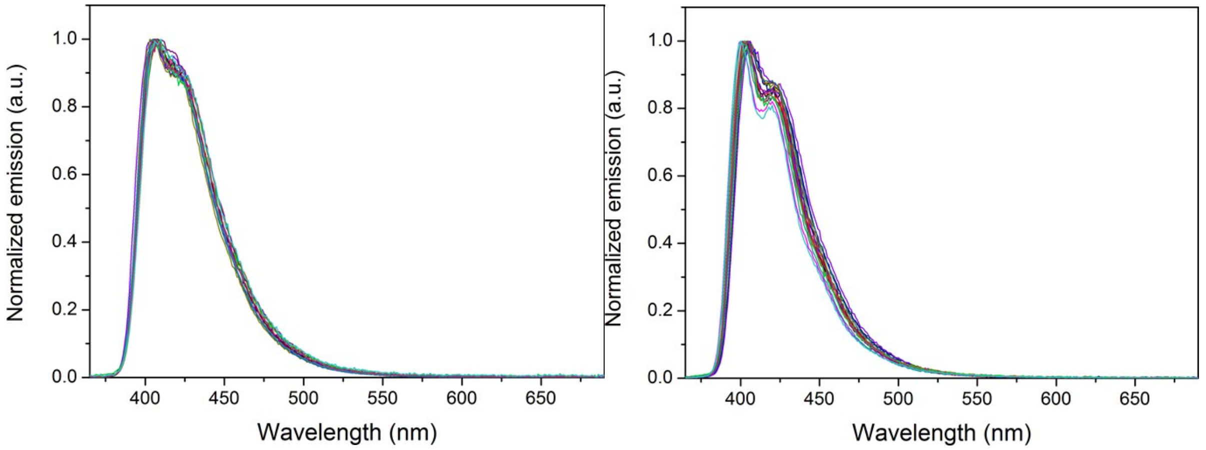

Figure S3. Normalized emission profile of pA in ssDNA (left) and dsDNA (right) with all possible combinations of A, G, C and T as nearest neighbors. Samples were prepared as described in the experimental section.

5.8. Figure S4

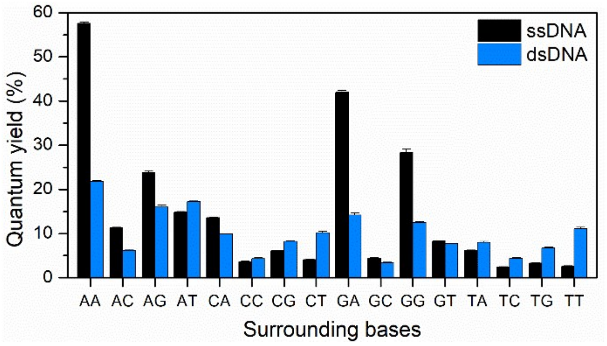

Figure S4. Fluorescence quantum yields with standard errors of pA in ssDNA and dsDNA. Letters on the x-axis denote the bases surrounding pA.

5.9. Figure S5

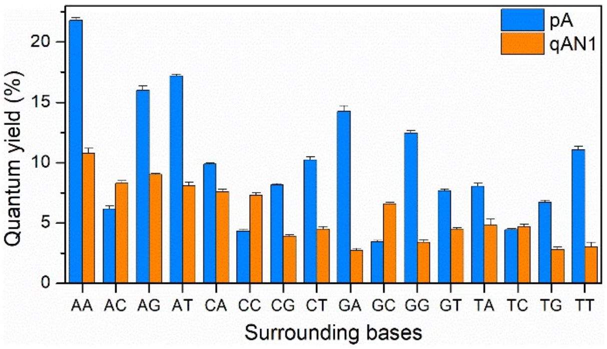

Figure S5. Fluorescence quantum yields with standard errors of pA in dsDNA compared with qAN1, one of the brightest adenine FBAs in DNA.[5] Letters on the x-axis denote the bases surrounding pA.

5.10. Table S3

Table S3. Mean lifetime of pA in single- and double-stranded DNA sequences.

NN[a]	Single strands <τ> (ns)[b]	Double strands <τ> (ns)[b]
AA	7.4 ±0.1	3.4 ±0.1
AC	1.5 ±0.1	1.1 ±0.1
AG	2.8 ±0.2	3.8 ±0.1
AT	1.9 ±0.2	3.1 ±0.1
CA	1.8 ±0.1	1.8 ±0.1
CC	0.49 ±0.04	0.82 ±0.03
CG	0.83 ±0.06	1.9 ±0.1
CT	0.58 ±0.06	1.7 ±0.1
GA	5.5 ±0.3	2.4 ±0.1
GC	0.55 ±0.05	0.71 ±0.02
GG	3.7 ±0.2	2.8 ±0.1
GT	1.2 ±0.1	1.6 ±0.1
TA	0.78 ±0.08	1.4 ±0.1
TC	0.39 ±0.04	0.88 ±0.04
TG	0.46 ±0.05	1.5 ±0.1
TT	0.37 ±0.03	2.0 ±0.1
Average	1.90	1.94

[a] Sequences were named according to the nucleosides surrounding pA; See Table 1. [b] Fluorescence decays were measured using a TCSPC setup with a laser diode emitting at 377 nm and emission collection at 408 nm. All decays were fitted to either bi-or triexponential functions. The reported fluorescence lifetimes are an average of two or more experiments reported with the standard error of the mean.

5.11. Table S4

Table S4. FRET-efficiencies determined using steady-state fluorescence and TCSPC measurements.

Sample	Separation (no. bp.)	FRET efficiency steady-state[a]	FRET efficiency lifetime[a]
D11A20	2	0.955 ±0.009	0.967 ±0.002
D11A19	3	0.949 ±0.010	0.964 ±0.010
D9A20	4	0.924 ±0.007	0.944 ±0.001
D9A19	5	0.856 ±0.004	0.868 ±0.002
D7A20	6	0.500 ±0.003	0.487 ±0.013
D7A19	7	0.172 ±0.007	0.136 ±0.006
D11A14	8	0.334 ±0.018	0.293 ±0.026
D11A13	9	0.396 ±0.026	0.370 ±0.011
D9A14	10	0.320 ±0.002	0.313 ±0.001
D9A13	11	0.146 ±0.021	0.132 ±0.020
D7A14	12	0.068 ±0.013	0.038 ±0.027
D7A13	13	0.070 ±0.037	0.050 ±0.022

[a] Values are reported with their 95% confidence intervals. Duplexes were formed as described in the experimental section, and were measured at RT in phosphate buffer, pH 7.5, 123 mM Na^+.

6. Two-Photon Excitation

A mode-locked Ti:sapphire laser (Coherent Mira pumped by Coherent Verdi), producing pulses of duration ~ 150 fs at a repetition rate of 76 MHz, was used as excitation source. A variable reflective neutral density filter was used to attenuate the excitation beam, which then passed through a dichroic mirror (Semrock Brightline FF735-Di02) and was focused by a 10× objective (Olympus) into the sample solution, contained in a 1 cm pathlength cuvette. Fluorescence emission was collected by the same objective, reflected from the dichroic mirror, passed through a shortpass filter (Semrock Brightline FF01-720/SP-25) and detected by a fibre-coupled spectrometer (Ocean Optics USB2000+), with an acquisition time of 2 s. The incident power was measured using a Coherent FieldMaster power meter. The pA base was dissolved in EtOH (99.9%, Fisher) and the pA-modified oligonucleotides in sodium phosphate buffer, as described above.
The two-photon absorption cross section (σ^2) of the sample was calculated relative to a reference standard, using equation S10.

$$\frac{\sigma_2^S \Phi^S}{\sigma_2^R \Phi^R} = \frac{\eta^R n^S C^R F^S \langle P^R \rangle^2}{\eta^S n^R C^S F^R \langle P^S \rangle^2} \qquad \text{(S10)}$$

where Φ is the fluorescence quantum yield, η is a term that accounts for the wavelength-dependent collection efficiency of the fluorescence, n is the refractive index of the solvent, C is the concentration, F is the integrated fluorescence signal from the recorded spectrum, P is the excitation power, and superscripts S and R refer to sample and reference, respectively.

The measurements were made at an excitation wavelength of 780 nm, against three different standards, Rhodamine 6G in MeOH (σ_2 = 70 GM; Φ = 0.93)[1], coumarin 153 in DMSO (σ_2 = 11 GM; Φ = 0.76)[1] and coumarin 153 in toluene (σ_2 = 14 GM; Φ = 0.87).[1] The emission intensities (*F*) of sample and reference standard were measured as a function of incident laser power *(P*), for at least 10 different laser powers, and the relative cross section was calculated using the ratio of the gradients of the respective plots of *F* versus P^2.
The two-photon cross sections have an estimated accuracy of ±10%, due to uncertainty in the cross-sections of the standards and errors in the measurement of the spectral throughput, absorption spectra and emission spectra.

6.1. Table S5

Table S5. Two-photon cross section, σ^2, and two-photon brightness, $\Phi\sigma^2$, for pA base measured relative to three reference standards, at excitation wavelength 780 nm.

Reference standard[a]	σ^2 (GM)	$\Phi\sigma^2$ (GM)[b]
Rhodamine 6G (MeOH)	6.8	5.4
Coumarin 153 (DMSO)	6.6	5.3
Coumarin 153 (toluene)	6.5	5.2
Average	**6.6 ± 0.5**	**5.3**

[a] From reference [5]. The error in the standard values of σ^2 is reported as 8%. [b] Using a quantum yield of 80%.

6.2. Table S6

Table S6. Two-photon cross section, σ^2, and two-photon brightness, $\Phi\sigma^2$, for pA-modified single-strand oligonucleotide GA, measured relative to two reference standards, at excitation wavelength 780 nm.

Reference standard[a]	σ^2 (GM)	$\Phi\sigma^2$ (GM)[b]
Rhodamine 6G (MeOH)	2.9	1.2
Coumarin 153 (DMSO)	3.0	1.3
Average	**3.0 ± 0.5**	**1.3**

[a] From reference [5]. The error in the standard values of σ^2 is reported as 8%. [b] Using a quantum yield of 42%.

6.3. Table S7

Table S7. Two-photon cross section, σ^2, and two-photon brightness, $\Phi\sigma^2$, for pA-modified double-strand oligonucleotide GA, measured relative to two reference standards, at excitation wavelength 780 nm.

Reference standard[a]	σ^2 (GM)	$\Phi\sigma^2$ (GM)[b]
Rhodamine 6G (MeOH)	2.5	0.35
Coumarin 153 (DMSO)	2.4	0.35
Average	**2.4 ± 0.5**	**0.35**

[a] From reference [5]. The error in the standard values of σ^2 is reported as 8% [b] Using a quantum yield of 14%.

7. References

[1] J. R. Lakowicz, *Principles of Fluorescence Spectroscopy*, 3rd ed., Springer, New York, **2006**.

[2] aK. Börjesson, S. Preus, A. H. El-Sagheer, T. Brown, B. Albinsson, L. M. Wilhelmsson, *J. Am. Chem. Soc.* **2009**, *131*, 4288-4293; bC. Carlsson, A. Larsson, M. Bjorkman, M. Jonsson, B. Albinsson, *Biopolymers* **1997**, *41*, 481-494.

[3] R. C. MacDonald, R. I. MacDonald, B. P. M. Menco, K. Takeshita, N. K. Subbarao, L.-r. Hu, *Biochimica et Biophysica Acta (BBA) - Biomembranes* **1991**, *1061*, 297-303.

[4] I. Pfeiffer, F. Höök, *J. Am. Chem. Soc.* **2004**, *126*, 10224-10225.

[5] M. S. Wranne, A. F. Füchtbauer, B. Dumat, M. Bood, A. H. El-Sagheer, T. Brown, H. Gradén, M. Grøtli, L. M. Wilhelmsson, *J. Am. Chem. Soc.* **2017**, *139*, 9271-9280.

[6] S. De Reguardati, J. Pahapill, A. Mikhailov, Y. Stepanenko, A. Rebane, *Opt. Express* **2016**, 24, 9053.
